# Orchestrating cancer therapy: Recent advances in nanoplatforms harmonize immunotherapy with multifaceted treatments

**DOI:** 10.1016/j.mtbio.2024.101386

**Published:** 2024-12-09

**Authors:** Rongwei Xu, Pei Lin, Jiarong Zheng, Yunfan Lin, Zizhao Mai, Ye Lu, Xu Chen, Zihao Zhou, Li Cui, Xinyuan Zhao

**Affiliations:** a Stomatological Hospital, School of Stomatology, Southern Medical University, Guangzhou, 510280, Guangdong, China; b Department of Dentistry, The First Affiliated Hospital, Sun Yat-Sen University, Guangzhou 510080, China; c School of Dentistry, University of California, Los Angeles, Los Angeles, 90095, CA, USA

**Keywords:** Nanoplatform, Immunotherapy, Synergistic effect, Cancer therapy, Oncology

## Abstract

Advancements in cancer therapy have increasingly focused on leveraging the synergistic effects of combining immunotherapy with other treatment modalities, facilitated by the use of innovative nanoplatforms. These strategies aim to augment the efficacy of standalone treatments while addressing their inherent limitations. Nanoplatforms enable precise delivery and controlled release of therapeutic agents, which enhances treatment specificity and reduces systemic toxicity. This review highlights the critical role of nanomaterials in enhancing immunotherapy when combined with chemotherapy, radiotherapy, photodynamic therapy, photothermal therapy, and sonodynamic therapy. Additionally, it addresses current challenges, including limited in vivo studies, difficulties in standardizing and scaling production, complexities of combination therapies, lack of comparative analyses, and the need for personalized treatments. Future directions involve refining nanoplatform engineering for improved targeting and minimizing adverse effects, alongside large animal studies to establish the long-term efficacy and safety of these combined therapeutic strategies. These efforts aim to translate laboratory successes into clinically viable treatments, significantly improving therapeutic outcomes and advancing the field of oncology.

## Graphical abstract

**Figure:**
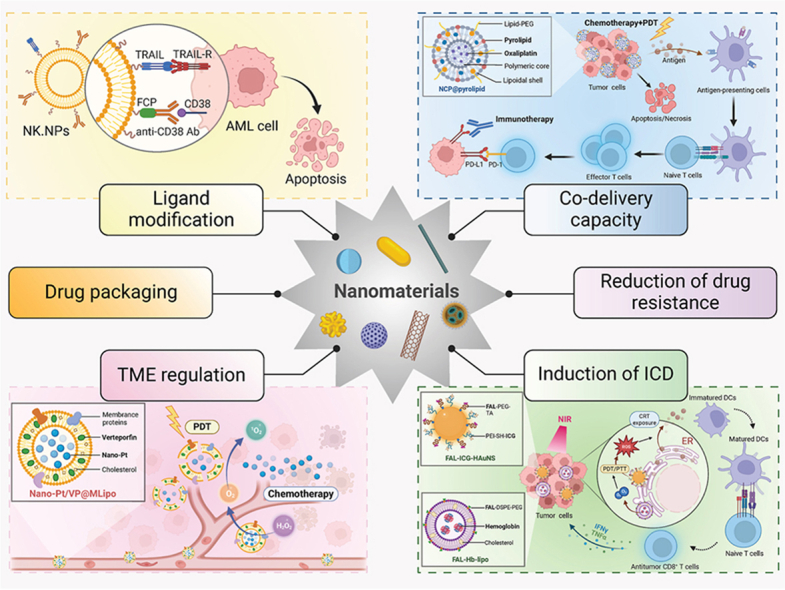

## 1 Introduction

Immunotherapy represents a transformative frontier in oncology, harnessing the body's immune system to combat cancer. This therapeutic modality capitalizes on the specificity and adaptability of the immune response, aiming to enhance its ability to recognize and eradicate cancer cells [1,2]. The strategies employed in immunotherapy range from checkpoint inhibitors, which release the brakes on T cells to allow them to attack cancer cells more effectively [3,4], to adoptive cell transfer, where T cells are engineered ex vivo for enhanced tumor targeting and then reintroduced into the patient [5]. Additional approaches include cancer vaccines that provoke immune responses against tumor-specific antigens and immunomodulators that tweak the immune environment to tilt the balance in favor of anti-tumor activity [6,7]. By reactivating the patient's immune surveillance, these therapies offer a potent means to achieve durable cancer control, potentially leading to sustained remission and improved patient outcomes.

The efficacy of immunotherapy is often limited by a combination of biological and clinical factors that can undermine its therapeutic potential. One of the primary challenges is the inherent heterogeneity of tumors, which include a diverse array of cancer cell subtypes within a single tumor. This variability not only complicates the immune system's ability to uniformly recognize and attack tumor cells but also fosters environments where some cancer cells can escape immune surveillance through mutations that alter antigenic profiles [8,9]. Additionally, the tumor microenvironment (TME) exerts a potent immunosuppressive effect. It often features a high concentration of regulatory cells such as regulatory T cells (Tregs) and myeloid-derived suppressor cells (MDSCs), which suppress effector T cells and natural killer cells (NK cells) that are crucial for anti-tumor immunity [10,11]. The TME also frequently expresses various immune checkpoint proteins, like PD-L1, which bind to receptors on immune cells to deactivate them, thus protecting tumor cells from immune attack [12]. Another limiting factor in immunotherapy efficacy is the presence of metabolic barriers within the TME, such as hypoxia and nutrient depletion, which can suppress the proliferation and function of infiltrating immune cells [13,14]. Additionally, patient-specific factors play a critical role; genetic variations can affect the expression and function of immune-related genes, influencing how patients respond to immunotherapy [15,16].

To overcome these challenges, the field of oncology is actively exploring combination therapies that integrate immunotherapy with other treatment modalities, such as chemotherapy, radiation, photodynamic therapy (PDT), photothermal therapy (PTT), and nanomaterial-based systems [17]. These combination strategies aim to modulate the TME, enhance tumor immunogenicity, and either restore or boost the immune response. Central to these strategies are nanoplatforms, which are transforming oncology by enhancing targeted drug delivery with high precision [[18], [19], [20], [21]]. These nanoscale platforms not only optimize therapeutic efficacy and minimize collateral damage by precisely targeting tumor cells and reducing systemic side effects, but they also enable the integration of multiple therapeutic agents and diagnostic markers. This facilitates the development of personalized medicine strategies, tailoring treatments to the specific genetic and molecular profiles of individual tumors.

This review explores the integration of immunotherapy with other treatment modalities using nanoplatforms to overcome the limitations of standalone immunotherapies. It begins by outlining the fundamental principles of synergistic interactions between immunotherapy and other therapies. The critical role of nanomaterials in facilitating these synergies is then highlighted. Importantly, we critically summarize and review the significant applications of various nanomaterials in combination with PDT, PTT, chemotherapy, radiotherapy (RT) and sonodynamic therapy (SDT), underscoring their potential to enhance immunotherapy efficacy, reduce side effects, and address TME challenges. Finally, the review addresses current research challenges and future directions, offering valuable perspectives and insights for innovation and advancement in cancer immunotherapy.

## 2 Mechanisms of the synergistic effect of immunotherapy and conventional cancer therapies

Chemotherapy has been shown to augment the efficacy of immunotherapy through several mechanistic pathways that modulate the TME. Primarily, chemotherapeutic agents induce immunogenic cell death (ICD), which is characterized by the release of damage-associated molecular patterns (DAMPs) such as ATP, HMGB1, and calreticulin [22,23]. These DAMPs facilitate dendritic cell (DC) recruitment and maturation, thereby enhancing antigen presentation and T cell priming. For instance, paclitaxel (PTX) triggers ICD-associated DAMPs via TLR4 signaling, activating NF-κB and promoting antitumor immunity [24]. Additionally, chemotherapy can modulate the expression of immune checkpoint molecules, thus sensitizing them to checkpoint inhibitors. Platinum-based chemotherapeutics reduce the expression of the T cell inhibitory molecule PD-L2 on human DCs and tumor cells, enhancing antigen-specific T cell responses and Th1 cytokine secretion [25]. Moreover, it can selectively deplete immunosuppressive cell populations, reducing local immunosuppression and fostering a more permissive environment for immune effector functions. For instance, PTX combined with ginsenoside metabolite compound K selectively depletes immunosuppressive Tregs and increases T helper cell 17, inducing cancer cell pyroptosis via the JAK-STAT pathway. In vivo studies confirm significant inhibition of lung cancer progression, highlighting this combination as a promising immunotherapy strategy [26].

RT, a conventional cancer treatment, has also been increasingly recognized for its synergistic potential with immunotherapy [27]. By inducing DNA damage and subsequent tumor cell death, RT exposes tumor neoantigens and releases immunostimulatory signals. This exposure not only enhances antigen presentation but also recruits and activates DCs, crucial for initiating T-cell responses [28]. RT upregulates immunogenic neoantigens in triple-negative breast cancer (TNBC), enhancing their presentation. Vaccination with these neoepitopes elicits CD8^+^ and CD4^+^ T cells, boosting immunotherapy by targeting irradiated cells and promoting Th1 cytokine production and epitope spread [29]. Furthermore, RT can modulate the expression of immune checkpoints on tumor cells or immune cells, making tumors more susceptible to checkpoint blockade therapies. For instance, RT upregulates PD-L1 expression in hepatocellular carcinoma (HCC) via the cGAS-STING pathway, leading to immune cloaking and reduced Cytotoxic T lymphocyte (CTL) activity. Combining RT with anti-PD-L1 therapy augments antitumor immunity, highlighting the synergy between RT and immunotherapy in HCC [30].

Targeted therapy has proven to be an effective complement to immunotherapy, enhancing its efficacy through several mechanistic pathways. By selectively inhibiting oncogenic pathways, targeted agents can modulate the TME to improve immune visibility and responsiveness [31]. Additionally, targeted therapies can reduce immunosuppressive factors within the TME, facilitating a more robust immune response. For instance, targeted therapy using an anti-WNT2 monoclonal antibody enhances Immune checkpoint inhibitors (ICI) efficacy in oesophageal squamous cell carcinoma and colorectal cancer by disrupting CAF-mediated immune evasion. Anti-WNT2 restores DC differentiation and antitumor T-cell responses via the SOCS3/p-JAK2/p-STAT3 signaling pathway, promoting antitumor immunity and enhancing ICI effectiveness [32].

Similarly, PDT and PTT have been recognized for their potential to enhance the efficacy of immunotherapy by inducing ICD and remodeling the TME. PDT involves the activation of photosensitizing agents by light to produce reactive oxygen species (ROS), which cause direct tumor cell damage and subsequent release of DAMPs [33]. For instance, PDT using aluminum-phthalocyanine in nanoemulsion (PDT-AlPc-NE) induces ICD in CT26 and 4T1 cancer cells, marked by the emission of DAMPs. PDT-treated cells enhance antitumor immunity in vivo, as shown in a vaccination-challenge model [34]. Additionally, PTT utilizes photothermal agents to convert light energy into heat, causing thermal destruction of tumor cells. This thermal ablation not only results in the release of tumor antigens but also induces a local inflammatory response that can overcome the immunosuppressive TME [35].

**Fig. 1:**
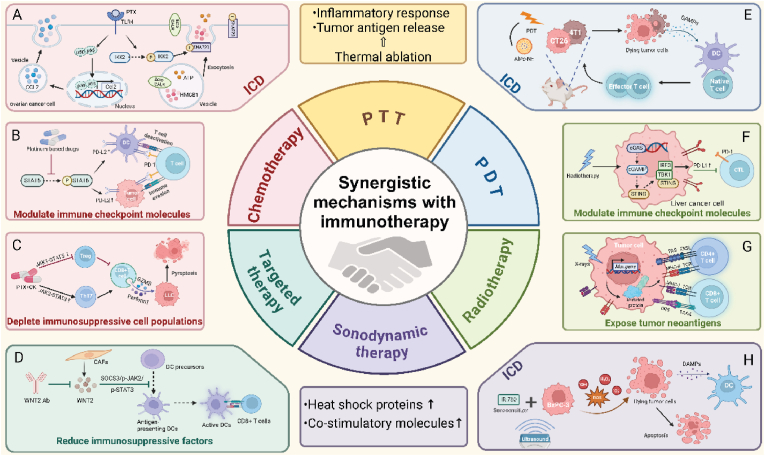
**Mechanisms of the synergistic effect of immunotherapy and conventional cancer therapies. (A)** PTX activates TLR4 signaling in ovarian cancer cells, leading to NF-κB-mediated transcription of pro-inflammatory cytokines and exocytotic release of ATP and HMGB1, inducing ICD. **(B)** Platinum-based drugs inhibit STAT6 phosphorylation, reducing PD-L1 expression on DCs and tumor cells. This leads to decreased T cell deactivation and enhances immune surveillance, countering tumor immune evasion. **(C)** Targeted therapy with PTX-CK disrupts JAK2/STAT3 signaling, enhancing CD8^+^ T cell cytotoxicity and reducing Treg suppression. This modulation fosters a pro-inflammatory environment conducive to tumor control by promoting the release of cytolytic proteins like granzyme B and perforin from CD8^+^ T cells. **(D)** WNT2 antibody therapy inhibits WNT2 signaling, impacting the SOCS3/p-JAK2/p-STAT3 pathway in CAFs and DC precursors. This leads to the activation of antigen-presenting DCs and subsequent stimulation of CD8^+^ T cell responses, enhancing immune surveillance against tumor cells. **(E)** PDT using the photosensitizer AlPc-NE targets CT26 and 4T1 tumor cells in a mouse model, resulting in ICD. The therapy triggers the release of damage- DAMPs from dying tumor cells, which stimulate DCs and subsequently activate effector T cells, enhancing anti-tumor immune responses. **(F)** Radiotherapy induces the cGAS-STING pathway in liver cancer cells, leading to increased expression of PD-L1 through activation of IRF3 and TBK1. This modulation facilitates immune evasion by tumor cells but also presents potential targets for enhancing the effectiveness of CTL-mediated cytotoxicity against tumor cells. **(G)** Radiotherapy exposes mutated proteins as neoantigens in tumor cells, enhancing their recognition by T cells. The process involves the upregulation of MHC molecules, facilitating the presentation of these neoantigens and promoting T cell-mediated tumor cell targeting through engagement of CD4^+^ and CD8^+^ T cells. **(H)** Sonodynamic therapy with the sonosensitizer IR-780 in BXPC-3 tumor cells generates ROS and leads to apoptosis. The dying tumor cells release DAMPs, which activate DCs to promote an adaptive immune response against the tumor.

SDT enhances the efficacy of immunotherapy by inducing ICD and modulating the TME [36]. When activated by ultrasound, sonosensitizers produce reactive ROS leading to tumor cell apoptosis and necrosis. This cell death releases tumor-associated antigens and DAMPs [37]. Moreover, SDT can increase the expression of heat shock proteins and co-stimulatory molecules, further boosting T-cell activation. The resultant enhanced antigen presentation and T-cell activity contribute to a more effective immunotherapeutic response, particularly in conjunction with checkpoint inhibitors. For instance, SDT using ultrasound-activated IR-780 induces significant ROS production and cancer cell death in BxPC-3 pancreatic cancer cells. This process triggers ICD and the release of DAMPs, promoting DC activation and an effective immune response (Fig. 1) [37]. In summary, combining conventional therapies—chemotherapy, radiotherapy, targeted therapy, PDT, PTT, and SDT—with immunotherapy enhances antitumor immunity by inducing ICD, modulating immune checkpoints, and reshaping the TME to favor immune activation. This multimodal strategy holds promise for overcoming immune resistance and achieving more durable cancer responses.

## 3 Nanomaterial-driven enhancements in synergistic cancer therapies

Nanomaterial-based systems stand at the forefront of innovative cancer treatment strategies, particularly due to their potential to significantly enhance the synergistic effects of combinative therapies [38]. These systems leverage the unique properties of nanomaterials to overcome several limitations associated with conventional cancer therapies. Firstly, nanomaterials, through their intrinsic immunomodulatory properties, hold substantial promise for cancer treatment by reshaping the TME and enhancing immune responses. For instance, the FDA-approved iron oxide nanoparticle ferumoxytol exhibits intrinsic anti-tumor effects by inducing pro-inflammatory M1 macrophage polarization in tumor tissues. In vitro, ferumoxytol-treated macrophages enhanced caspase-3 activity in adenocarcinoma cells and promoted Th1-type responses. In vivo, ferumoxytol nanoparticles inhibited tumor growth and prevented liver metastasis, highlighting their potential ''off-label'' application in cancer immunotherapy through macrophage modulation [39]. In addition, silica nanoparticles (SiNPs) exhibit intrinsic anti-tumor effects by promoting M1 macrophage polarization within the lung TME. By activating the NF-κB pathway and glycolytic mechanisms, SiNPs suppress lung cancer progression and modulate pre-metastatic and metastatic niches [40]. Notably, gold nanoparticles (AuNPs) exhibit intrinsic therapeutic properties in cancer therapy by selectively disrupting NLRP3 inflammasome signaling in macrophages through ROS scavenging, thus reducing IL-1β production. When conjugated with the MDSC-targeting peptide H6, AuNPs effectively decrease MDSC populations and IL-1β levels within the TME, enhancing T cell activation and improving immunotherapy outcomes in PD-1-sensitive and -resistant tumor models [41]. Moreover, titanium dioxide NPs (TiO₂ NPs) inherently induce a pro-inflammatory response, activating M1 macrophages and amplifying cytokine production, which correlates with inhibited cancer metastasis. In vivo, TiO₂ NPs trigger immune cell infiltration and systemic inflammation in liver and spleen, demonstrating their potential to modulate immune activity against metastasis through intrinsic immunostimulatory properties [42]. In conclusion, these findings underscore the significant role of nanomaterials' intrinsic immunomodulatory properties in revolutionizing cancer treatment strategies, fueling the development of nanomaterial-driven synergistic cancer therapies.

**Fig. 2:**
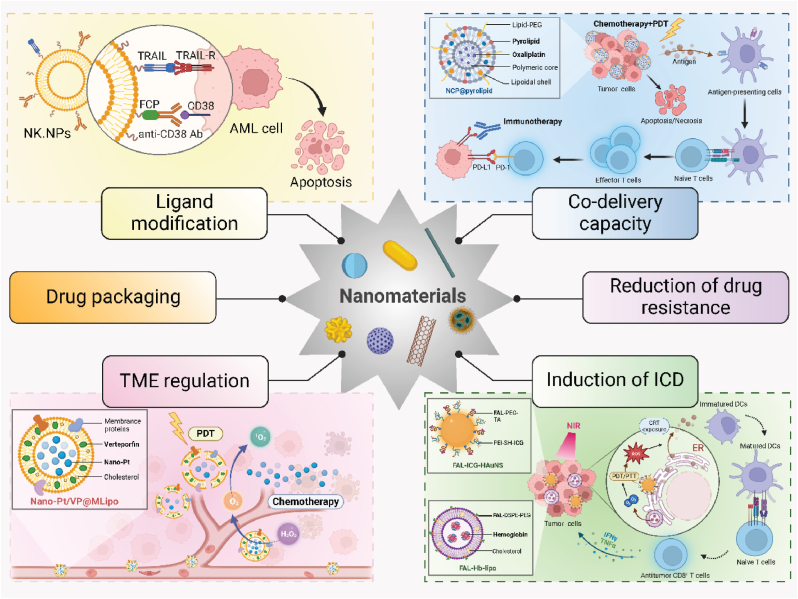
**Nanomaterial-driven enhancements in synergistic cancer therapies.** Nanomaterials enhance the synergy of combined cancer therapies through targeted ligand modification, efficient drug packaging, TME regulation, co-delivery capacity, reduction of drug resistance and induction of ICD. By engineering nanoparticles to specifically target cancer cells with apoptosis-inducing ligands and deliver multiple therapeutic agents directly to the tumor site, these strategies reduce drug resistance and improve treatment efficacy. Additionally, nanoparticles facilitate the induction of ICD and precisely regulate the TME. These approaches optimize local conditions for therapies such as PDT and chemotherapy, bolstering the immune response and disrupting immunosuppressive barriers within the TME, significantly enhancing therapeutic outcomes.

Secondly, nanomaterials can be engineered to target tumor cells specifically, minimizing off-target effects and enhancing therapeutic efficacy. Their surface can be modified with ligands that recognize and bind to receptors overexpressed on tumor cells, ensuring that the active agents are delivered precisely where needed. For instance, engineered NK cell-mimic nanoparticles (NK.NPs) incorporate death ligand TRAIL and CD16 peptide for antibody-mediated tumor recognition. Functionalized with anti-CD38 antibody, NK.NPs effectively targeted and killed CD38-positive acute myeloid leukemia (AML) cells in vitro, ex vivo, and in vivo, reducing AML burden in bone marrow [43]. Additionally, nanocarriers can encapsulate drugs, protecting them from premature degradation and releasing them in a controlled manner at the tumor site. This targeted delivery is crucial for therapies like chemotherapy, where reducing systemic toxicity is a primary concern [44]. Thirdly, nanomaterials are uniquely capable of carrying multiple types of therapeutic agents simultaneously. This co-delivery capability allows for the direct combination of different therapeutic modalities within a single platform. For instance, nanoscale coordination polymer core-shell nanoparticles, encapsulating oxaliplatin and pyropheophorbide-lipid conjugate (pyrolipid), enhance the efficacy of PD-L1 antibody-mediated immunotherapy in advanced colorectal cancer [45]. Fourthly, the TME often presents a significant barrier to effective cancer treatment. Nanomaterials can alter the TME to make it more conducive to therapy. Nano-Pt/VP@MLipo liposomes leverage platinum nanoparticles to catalyze oxygen production, altering the TME by reducing hypoxia. This enhances verteporfin's photodynamic effect and nanoparticle penetration, significantly improving treatment efficacy against tumor growth and metastasis [46]. Fifthly, nanomaterials used in PDT and PTT can induce ICD, which releases tumor antigens in a context that is conducive to immune system activation. This not only helps in the direct killing of cancer cells but also turns the tumor itself into a vaccine against itself, promoting an adaptive immune response that can target tumor cells systemically. For instance, ER-targeting nanosystem combining FAL-ICG-HAuNS and hemoglobin liposomes enhances photodynamic and photothermal therapies, inducing ER stress and calreticulin exposure under near-infrared (NIR) light. This activation stimulates DC function and CD8^+^ T cell responses, boosting ICD and anti-tumor efficacy through ROS-mediated mechanisms [47]. Lastly, the multi-modal nature of nanomaterial-based systems can help overcome mechanisms of drug resistance. Nanoparticles can bypass cellular efflux pumps, a common resistance mechanism in chemotherapy, ensuring higher intracellular concentrations of chemotherapeutics [48]. Moreover, the combination of different treatment modalities can attack cancer cells via multiple pathways simultaneously, reducing the likelihood of developing resistance (Fig. 2).

Accumulating preclinical evidence underscores the efficacy of diverse nanoplatforms in enhancing cancer therapy. Through precise targeting, improved pharmacokinetics, and reduced systemic toxicity, these platforms demonstrate significant therapeutic potential across various tumor models, offering a robust foundation for their potential clinical application. For instance, mitochondria-targeting nanoparticles enhance PDT efficacy in breast cancer by conserving local oxygen within tumors. Conjugating 5-aminolevulinic acid to triphenylphosphine and coating with folic acid enables precise mitochondrial delivery. In BALB/c mice with MCF-7 xenografts, these nanoparticles improve PDT effectiveness, confirmed by pharmacokinetic and distribution studies, underscoring their potential to optimize PDT under anoxic conditions [49]. In addition, macrophage membrane-coated, biodegradable camptothecin nanoprodrugs enhanced tumor accumulation and suppressed breast cancer growth and metastasis in preclinical murine models. Leveraging macrophage cloaking and polymer conjugation, this bioadhesive nanoparticle formulation showed high tumor specificity, improved pharmacokinetics, and minimal systemic toxicity, underscoring its potential to improve cancer therapeutic efficacy [50]. Moreover, in preclinical models of colorectal cancer, *ALKBH5* mRNA-loaded exosome-liposome hybrid nanoparticles with folic acid modification significantly inhibited tumor progression by modulating the ALKBH5/JMJD8/PKM2 axis and reducing glycolysis. This nanoparticle system, tested in colitis-associated tumor models, effectively decreased tumorigenesis, demonstrating the therapeutic potential of targeting m^6^A modifications for colorectal cancer treatment [51]. Similarly, in an orthotopic osteosarcoma murine model, keratin nanoparticles co-encapsulating Chlorin e6 (Ce6) and PTX achieved a 78 % tumor reduction with combined photodynamic and chemotherapy significantly outperforming PTX monotherapy's 32 % reduction. These optimized parameters for light delivery, dosing, and treatment scheduling demonstrate the efficacy of nanoplatform-based combination therapy in osteosarcoma, advancing its potential for clinical translation [52]. Notably, glycol chitosan nanoparticles (CNPs) showed varied efficacy across pancreatic tumor models, highlighting the impact of TME differences on nanoparticle accumulation. Using non-invasive imaging, CNP targeting efficiency was assessed in BxPC3, PDC, and PDX models, revealing that vessel density, collagen, and hyaluronic acid content influenced CNP delivery. PDX models reflected inter-patient TME heterogeneity, suggesting they may better predict clinical permeation and retention effects, providing critical insights for personalized nanoparticle-based cancer therapies [53]. Collectively, nanomaterial-based systems represent a transformative approach in cancer therapy, enhancing the precision, efficacy, and synergy of combinative treatments. By enabling targeted delivery, co-encapsulation of therapeutic agents, modulation of the TME, and induction of ICD, these nanosystems address critical limitations of conventional therapies. Their multi-modal functionality offers a promising avenue to overcome drug resistance, potentially leading to more effective and durable anticancer responses.

## 4 Enhancing the synergistic effect of PDT and immunotherapy with nanotherapeutic platforms

### 4.1 Organic-based nanomaterials

Organic nanoparticle-based PDT has recently been shown to significantly increase the efficacy of immunotherapy in cancer treatment. For instance, graphdiyne oxide (GDYO) used in PDT increases the mechanical stiffness of oral squamous cell carcinoma cells, enhancing CTL responses and inflammatory cytokine production [54]. Additionally, a charge-reversal polymer nano-modulator (SPDMC N), designed for PDT, exploits an acidic TME to enhance tumor penetration and release the immunomodulator demethylcantharidin. Upon NIR photoirradiation, SPDMC N facilitates tumor ablation and DC maturation while demethylcantharidin modulates the CTL/Treg ratio, significantly inhibiting tumor growth and metastasis [55].

### 4.2 Hybrid organic-inorganic nanomaterials

Similarly, various types of hybrid organic-inorganic nanomaterials have been developed to enhance PDT efficacy, which in turn amplifies the effectiveness of immunotherapy. For instance, a novel immunotherapeutic nanobooster, C9SN, combines a glutaminase inhibitor with Ce6 to potentiate PDT against tumors. By inhibiting glutamine metabolism, C9SN prevents glutathione from neutralizing ROS, enhancing cell death and ICD effects. This dual-action not only promotes DC maturation and CTL activation but also repolarizes M2-type macrophages to M1-type, effectively targeting both primary and distant tumors [56]. Similarly, polyethylenimine hybrid thin shell hollow mesoporous silica nanoparticles (THMSNs), encapsulating Ce6, synergize photodynamic and immunotherapy. Enhancing cellular uptake and endosomal escape, these nanoparticles increase PDT efficiency, facilitating DC maturation and robust CD8^+^ T cell activation [36]. Interestingly, a nuclear-targeted photodynamic nanostrategy using mSiO_2_-Ion@Ce6-NLS nanoparticles enhances intratumoral enrichment and directs therapy to the nucleus. This approach triggers ICD and activates macrophages and DCs, facilitating CD8^+^ T-cell recruitment and strengthening antitumor immunity [57].

### 4.3 MOF-based nanomaterials

MOFs are being employed to improve the synergistic effects of PDT and immunotherapy. Notably, a metal-organic frameworks-based nanoagonist, DZ@A7, is engineered for tumor-specific, NIR light-enhanced delivery, activating the cGAS-STING pathway with minimized off-target effects. Under NIR irradiation, it generates ROS, releases mitochondrial DNA, and triggers zinc ion overload, enhancing cGAS activity and boosting DC maturation and T cell infiltration [58]. Additionally, copper (II) metalated MOF nanosheets enhanced with platinum nanoparticles and folate (Cu-TCPP(Al)-Pt-FA) deliver targeted, dual-enhanced PDT and mitigate tumor hypoxia. This nanomedicine exploits copper's GSH depletion and platinum's catalase-like activity, boosting ROS levels for effective PDT and triggering ICD. The approach not only amplifies oxidative stress and systemic immune response but also facilitates the transition of M2 to M1 macrophages, enhancing CTL infiltration and demonstrating significant antitumor effects [59].

### 4.4 Cell- and bacteria-hitchhiking nanomaterials

Cell- and bacteria-hitchhiking nanomaterials offer a versatile approach for enhancing combination cancer therapies, enabling precise targeting and synergistic activation of phototherapy, chemotherapy, and immunotherapy within the TME. For instance, red blood cell (RBC)-mimicking nanoparticles (M@AP) enhance tumoral photodynamic-immunotherapy by combining RBC membrane encapsulation with aggregation-induced emission luminogen (AIEgen) for ROS generation and Poly(I) as an immune stimulant. Enriched in tumors via the enhanced permeability and retention effect and in the spleen through RBC homing, M@AP nanoparticles induce ROS under light irradiation, releasing tumor antigens and stimulating immune responses [60]. Notably, cancer cell membrane-coated nanoparticles (CCMV/LTNPs) enhance photoimmunotherapy by integrating PDT with TLR7 agonists and tumor antigens. These biomimetic nanoparticles specifically kill tumor cells via ROS generation from PDT, while the immune adjuvant and cancer cell membrane-derived antigens activate host immune responses to target residual cells [61]. Additionally, an *E. coli* Nissle 1917-based biohybrid (EcN@TTVP) loaded with aggregation-induced emission photosensitizer (AIE-PS) enhances Cerenkov radiation-induced PDT and activates anti-tumor immunity. Targeting tumors with both EcN@TTVP and radiopharmaceutical 18F-FDG triggers ICD and DC maturation, priming CTLs [62]. This synergistic approach achieves effective tumor remission with minimal toxicity, presenting a promising method for deep-seated tumor treatment.

### 4.5 Functional composite nanosystems

Recently, multifunctional composite nanosystems have been developed to boost cooperative enhancement in PDT and immunotherapy. ATO/PpIX-SMN nanocubes enhance protoporphyrin IX-mediated PDT in TNBC by facilitating ICD and downregulating Wnt/β-catenin signaling. Combined with anti-PD-L1, these nanocubes promote DC maturation, increase CTL infiltration, and reduce Tregs, effectively improving treatment efficacy against primary and distal tumors [63]. Similarly, the MB@MSP nanocarrier, integrating a D-peptide antagonist and methylene blue within a TME-responsive framework, utilizes MMP-2 and glutathione sensitivity for controlled drug release. This platform enhances tumor penetration, facilitates immune checkpoint blockade (ICB), and strengthens PDT-induced CTL responses [64]. Notably, Cu3P/1-MT nanocomposites combine PDT and immunotherapy, enhancing tumor ablation and ICD while modulating the immunosuppressive microenvironment. These nanocomposites effectively promote DC maturation and CD8^+^ T cell infiltration, and reduce Tregs and M2 macrophages, significantly amplifying antitumor responses in photothermal-immunotherapy contexts [65].

**Fig. 3:**
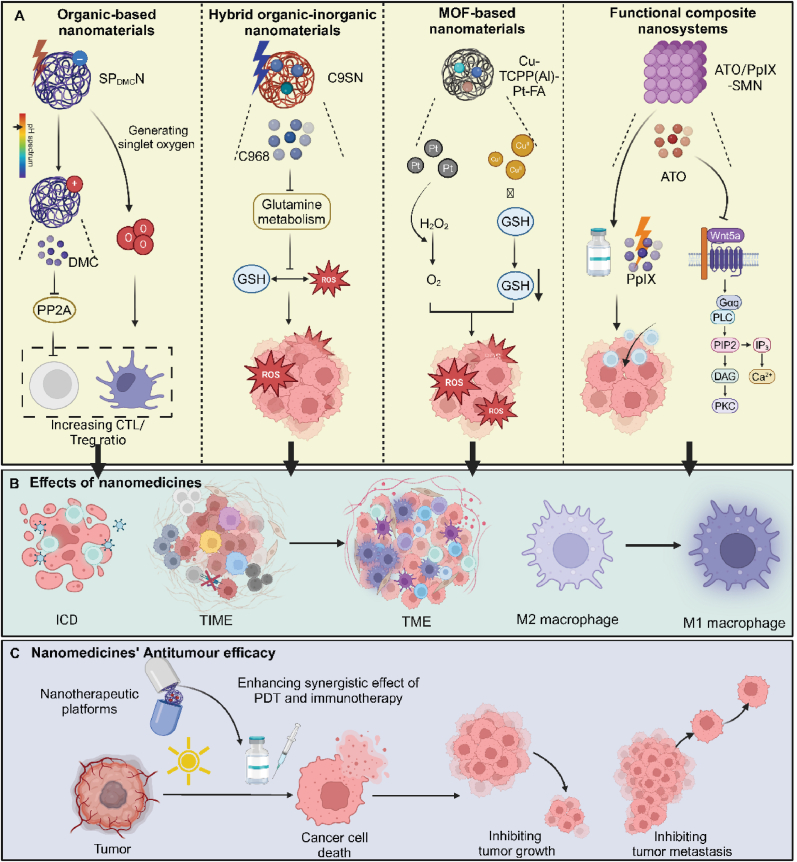
**Multifaceted role of nanomaterials in enhancing PDT and Immunotherapy synergy.** Nanomaterials serve as a pivotal component in advancing the synergistic effects of PDT and immunotherapy. Organic-based nanomaterials such as SP_DMC_N facilitates the generation of singlet oxygen, pivotal in activating downstream immune responses, including the modulation of the CTL/Treg ratio favorable for immune activation. Hybrid organic-inorganic nanomaterial C9SN target glutamine metabolism pathways, escalating ROS production, and further enhancing the immunogenic environment within tumors. MOF-based nanomaterial Cu-TCPP(Al)-Pt-FA catalyzes the conversion of intratumoral hydrogen peroxide to oxygen, mitigating hypoxia and boosting ROS levels to potentiate immune responses and PDT efficacy. Functional composite nanosystems, incorporating agents like ATO and PplX, directly impact signaling pathways such as Wnt5a and PLC, crucial for sustaining cellular immune responses and enhancing the overall antitumor efficacy. Through these diverse mechanisms, nanomaterials optimize both TME manipulation and immune system engagement, substantially improving the combined therapeutic outcomes of PDT and immunotherapy.

Importantly, various drugs, small molecule compounds, and antigens are loaded into composite nanosystems to enhance the combined effects of PDT and immunotherapy. For instance, a self-assembled nanomedicine, CCXB, combines Ce6 and celecoxib to deliver PDT that effectively reduces breast cancer cell proliferation and mitigates immunosuppressive environments. This approach enhances ICD, lowers PGE2 synthesis, and adjusts T cell populations, significantly amplifying in vivo antitumor immunity and reducing metastasis [66]. Similarly, a supramolecular prodrug nanoplatform co-delivers a photosensitizer and a bromodomain-containing protein 4 inhibitor (BRD4i) JQ1, targeting pancreatic cancer. The nanoparticles, formed by host-guest complexation using cyclodextrin-grafted hyaluronic acid, enhance PDT's immunogenicity and limit immune evasion by modulating key glycolysis and evasion regulators [67]. Likewise, a chimeric peptide, PpIX-1MT, forms nanoparticles that deliver a photosensitizer PpIX and an ICI 1 MT to tumors, utilizing a caspase-responsive linkage for cascaded drug release. Upon light irradiation, these nanoparticles generate ROS, inducing apoptosis and tumor antigen production, followed by enhanced immune response through the release of 1 MT. This innovative approach effectively activates CD8^+^ T cells, significantly inhibiting primary and metastatic tumors [68]. In addition, the development of PheoA-PEI/OVA nanoparticles, which incorporate a photosensitizer and a model antigen, enhances antigen-specific CD8^+^ T cell responses via photochemical internalization. These nanoparticles improve endocytosis, ROS generation, and cytosolic antigen release in DCs upon light stimulation, leading to increased T cell activation and notable tumor growth inhibition in vivo (Table 1) (Fig. 3) [69].

**Table 1:** Nanoplatforms for enhancing the synergistic effect of PDT and immunotherapy.

Nanoplatform	Composition	Mechanisms	Immune effects	Outcomes	Ref.
GDYO	Graphdiyne oxide	Shifting mechanical forces of OSCC cells	T-cell cytotoxicity↑, inflammatory cytokine secretion (IFN-γand TNF-α)↑	Enhancing antitumor immunotherapeutic effect	[54]
SP_DMC_N	Demethylcantharidin, photodynamic polymer	Inhibiting PP2A activity, enhancing tumor penetration	DC maturation↑, Tregs differentiation↓	Inhibiting primary and distant tumors in living mice	[55]
C9SN	C968, Chlorin e6 (Ce6)	Amplifying intracellular oxidative stress, remodeling ITM by blocking glutamine metabolism	DC maturation↑, recruiting and activating CTLs↑, M2-type TAMs↓, M1-type TAMs↑	Suppressing primary and distant tumors	[56]
Ce6@THMSNs	Ce6, polyethylenimine (PEI), thin shell hollow mesoporous silica NPs (THMSNs)	Releasing tumor-associated antigens	DC maturation↑, CD8^+^ CTLs activation↑	Obliterating primary tumors and inducing persistent tumor-specific immune responses, preventing distant metastasis	[184]
mSiO_2_-Ion@Ce6-NLS	Hydrogen Ce6, ionic liquid silicon (Ion), nuclear localization signal peptide (NLS: PKKKRKV), mSiO_2_	Facilitating release of damaged double-stranded DNA from tumor cells, stimulating interferon gene signaling	Activating macrophages↑, recruiting CD8^+^ T-cells↑	Inhibiting tumor growth and recurrence	[57]
DZ@A7	cGAS-STING nanoagonist, metal-organic frameworks	Generating mitochondria-targeted ROS, releasing mtDNA, inhibiting repair of nuclear DNA	Activating cGAS-STING pathway↑, DC maturation↑, CTLs infiltration↑	Eradicating primary tumor, establishing a long-term anti-tumor immunity, suppressing tumor metastasis	[58]
Cu-TCPP(Al)-Pt-FA	Cu-TCPP(Al), Pt NPs, folate	Enhancing ROS concentration for dual-enhanced PDT, amplifying oxidative stress	ICD↑, stimulating APCs↑, CTLs infiltration↑, M2 macrophage↓, M1 phenotype↑	Enhancing antitumor effect of PDT and immunotherapy	[59]
ATO/PpIX-SMN	Atovaquone (ATO), protoporphyrin IX (PpIX)	Enhancing PpIX-mediated PDT, downregulating Wnt/β-catenin signaling	DC maturation↑, CTLs infiltration↑, reducing Tregs↓	Treating primary and distal tumors	[63]
MB@MSP	PDPPA-1, methylene blue	Blocking immune checkpoint	Suppression of tumor metastasis effect of CD8^+^ CTLs↑	Enhancing therapeutic efficacy of combined treatment of metastatic tumors	[64]
Cu_3_P/1-MT	Indoleamine 2, 3-dioxygenase-1 inhibitors (1-MT), copper (I) phosphide	Promoting TAAs release, inhibiting the indoleamine 2, 3-dioxygenase-1	DC maturation↑, antigen presentation↑, CD8^+^ T cells infiltration↑, Tregs and M2 macrophages↓	Amplifying the antitumor therapeutic efficiency	[65]
CCXB	Ce6, celecoxib (CXB)	Triggering stronger PDT, mediating cyclooxygenase 2 (COX-2) inhibition to decrease synthesis of prostaglandin E2 (PGE2)	CTLs↑, Tregs↓	Inhibiting the metastasis of breast cancer	[66]
HCJSP	JQ1, photosensitizer, HA-CD, adamantine-conjugated heterodimers of pyropheophorbide a (PPa)	Recognizing highly expressed CD44 on the surface of pancreatic tumors, inhibiting expression of c-Myc and PD-L1, ICD	CTLs infiltration↑, activated T cells↑	Enhancing photoimmunotherapy of the pancreatic cancer	[67]
PpIX-1MT-Asp-Glu-Val-Asp (DEVD)	PpIX, 1 MT, caspase-responsive peptide sequence DEVD	Producing ROS, inducing apoptosis of cancer cells, enhancing caspase-3 and production of tumor antigens,	Activating CD8^+^ T cells↑	Inhibiting both primary and lung metastasis tumor	[68]
PheoA-PEI/OVA	Polyethylenimine PheoA-PEI, ovalbumin (OVA)	Increasing ROS, enhancing cytosolic antigen release	CD3^+^CD8^+^ T lymphocytes↑	Inhibiting E.G7 tumor growth in mice	[69]

Nanotherapeutic platforms markedly enhance the synergy of PDT and immunotherapy by facilitating targeted delivery, modulating immune responses, and amplifying antitumor efficacy. Leveraging a variety of nanomaterials—from organic and hybrid structures to MOFs and functional composites—these systems induce ICD, reprogram the TME, and activate adaptive immunity. This integrative approach represents a promising advancement for combinatorial cancer therapies, providing a versatile and potent framework for future treatment strategies.

## 5 Enhancing the synergistic effect of PTT and immunotherapy with nanotherapeutic platforms

### 5.1 Organic-based nanomaterials

Organic-based nanomaterials are used to enhance the synergistic effect of PTT and immunotherapy due to their biocompatibility, biodegradability, and the ease with which they can be functionalized with various therapeutic agents, allowing for precise tumor targeting, efficient heat generation, and robust immune system activation. For instance, a novel nanosystem utilizing reduced graphene oxide loaded with mitoxantrone and SB-431542, and activated by NIR laser, synergizes PTT, chemotherapy, and immunotherapy. This approach increases cytotoxic CD8^+^ T cell infiltration while reducing Tregs, offering a promising strategy for metastatic cancer therapy [70]. Additionally, polydopamine nanoparticles (PDMN-JQ1) provide a novel, multifaceted approach for TNBC treatment by synergizing targeted therapy, PTT, and immunotherapy. Loaded with JQ1, PDMNs down-regulate PD-L1 and inhibit the BRD4-c-MYC axis, enhancing CTL activation and inducing a durable immune-memory effect [71]. Interestingly, cuttlefish ink-derived nanoparticles (CINPs), rich in melanin and biocompatible components, synergize PTT and immunotherapy. Upon NIR irradiation, CINPs efficiently convert to heat, promoting tumor cell apoptosis and antigen release. Concurrently, these nanoparticles reprogram tumor-associated macrophages from an M2-like suppressive to an M1-like antitumor phenotype, enhancing CTL recruitment and activity [72].

### 5.2 Hybrid organic-inorganic nanomaterials

Hybrid organic-inorganic nanomaterials are employed to enhance the synergistic effect of PTT and immunotherapy due to their ability to integrate the high thermal conversion efficiency and stability of inorganic components with the biocompatibility and functional adaptability of organic materials, leading to precise tumor targeting, effective heat production, and strong immune system activation. A titanium carbide MXene-based nanoplatform linked with Mn^2+^-contained ovalbumin activates both innate and adaptive immunities upon NIR laser irradiation. This activation leads to efficient antigen presentation and DC maturation, significantly enhancing CTL infiltration and antitumor efficacy [73]. In addition, a NIR responsive nanoenzyme, PB-Mn/OVA NE, was developed by doping manganese into Prussian blue nanoparticles templated with ovalbumin. This nanoenzyme alleviates tumor hypoxia, enhances photothermal tumor ablation, and triggers ICD. It effectively activates the cGAS-STING pathway, enhancing DC function and boosting cytotoxic and memory T cell responses, thereby offering a comprehensive strategy for photothermal and immunotherapy of tumors [74]. Moreover, a photothermally activatable polymeric pro-nanoagonist (APNA) uses NIR-II light for precise spatiotemporal control in cancer immunotherapy. This nanoparticle, constructed from a NIR-II semiconducting transducer and an immunostimulant linked by a thermolabile linker, triggers localized tumor ablation and releases the immunostimulant upon NIR-II irradiation. The result is enhanced cytotoxic and helper T cell infiltration across distant tumors and organs, effectively inhibiting metastasis [75]. Notably, combining mesoporous organosilica nanoparticles loaded with Fe^3+^ and indocyanine green, and mild PTT efficiently induces oxidative cell death and DC activation in vitro. In vivo, it promotes T cell infiltration and robust adaptive immunity, demonstrating significant potential in TNBC treatment without notable side effects [76].

### 5.3 Metal-based nanomaterials

Metal-based nanomaterials are used to enhance the combinative effects of PTT and immunotherapy because their excellent light absorption and conversion efficiency allow for the effective generation of localized heat upon NIR irradiation, leading to precise tumor ablation and minimal damage to surrounding healthy tissues. For example, cerium oxide end-deposited gold nanorods (CEG) enhance NIR photoimmunotherapy by generating heat and ROS under NIR light, triggering ICD and boosting CTL infiltration in TNBC. When combined with PD-1 antibody, CEG reverses the immunosuppressive environment, amplifying the immune response and demonstrating effective synergy in TNBC treatment [77]. Similarly, a supramolecular cationic gold nanorod combines CRISPR/Cas9-mediated PD-L1 disruption with NIR-II light-induced mild hyperthermia for enhanced ICB therapy. This dual-action nanoplatform triggers ICD and promotes dendritic-to-T cell conversion, enhancing CTL infiltration and reprogramming the TME [78]. Additionally, the Au@Pt-based nanosystem, conjugated with an MMP-activated peptide, enhances cancer treatment by combining PTT and immunotherapy. This dual-function nanosystem effectively delivers photothermal heat and a D-peptide antagonist of PD-L1, promoting robust tumor ablation and CTL activation [79]. Moreover, AuPtAg-GOx nanomodulators are engineered to enhance immune responsiveness by integrating mild PTT and glucose oxidase-induced starvation. Upon laser irradiation, these nanomodulators stimulate T cell activation and modulate the TME, while their nanozyme activity reduces hypoxia to boost overall treatment efficacy. When combined with PD-L1 antibodies, this multifunctional platform significantly amplifies antitumor effects, demonstrating a novel method to combat immunologically "cold" tumors through a synergistic approach [80].

### 5.4 Cell- and bacteria-hitchhiking nanomaterials

Interestingly, due to their inherent ability to selectively target tumors, deliver therapeutic agents, and uniquely interact with the TME, bacteria are now being engineered into composite nanosystems to significantly enhance therapeutic outcomes. An engineered microbial nanohybrid combining *Escherichia coli* with Cu_2_O nanoparticles targets colon tumors, converting to Cu_x_S under high H_2_S conditions and exhibiting effective photothermal activity. This hybrid triggers ferroptosis and cuproptosis, reversing immunosuppression and enhancing DC and CD8^+^ T cell responses, particularly when paired with ICI, showing promise for precise photothermal-enhanced tumor therapy [81]. Similarly, decorating bacteria with triple immune nanoactivators, this approach employs polydopamine nanoparticles to conjugate tumor-specific antigens and checkpoint blocking antibodies on bacterial surfaces. Polydopamine facilitates photothermal macrophage repolarization, antigen-induced DC maturation, and checkpoint blockade-enhanced T cell activation, offering a multimodal, durable cancer immunotherapy platform with potent tumor-specific effects [82].

Alongside bacterial systems, various cell membranes also serve as valuable carriers for nanomaterials in therapeutic applications. For instance, a biomimetic nanoparticle coated with a microglia membrane (BM@MnP-BSA-aPD-1) effectively crosses the blood-brain barrier to target the immunosuppressive TME in glioblastoma. Leveraging the membrane's targeting capabilities, this nanoplatform combines metalloimmunotherapy and PTT to activate interferon pathways, induce ICD, and inhibit PD-1/PD-L1 signaling, thereby revitalizing antitumor immunity [83]. Similarly, hybrid cell-membrane-coated nanoparticles (Fe_3_O_4_-ICG@IRM), combining ovarian cancer cell and RBC membranes, offer precise targeting and prolonged circulation for ovarian cancer therapy. Using PTT, these biomimetic nanoparticles induce tumor antigen release, activating CD8^+^ T cells and reducing Foxp3^+^ Tregs. This synergistic approach enhances both primary and metastatic antitumor immune responses, showing promise for effective photothermal-immunotherapy in ovarian cancer [84].

### 5.5 Functional composite nanosystems

**Fig. 4:**
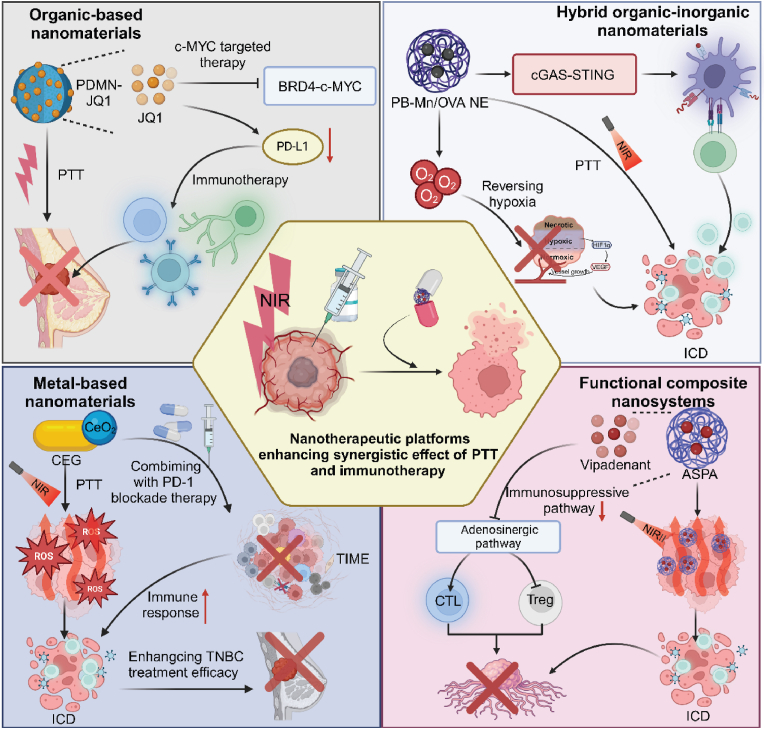
**Nanomaterial-enhanced PTT as a catalyst for immunotherapy synergy.** Nanomaterials critically enhance the synergistic effects of PTT and immunotherapy across multiple platforms. Metal-based nanomaterial CEG, combined with PD-1 blockade therapy, amplify the immune response and enhance treatment efficacy against TNBC through the generation of ROS and induction of ICD. Organic-based nanomaterial PDMN-JQ1, carrying c-Myc targeted therapies like JQ1, modulate immunological profiles by reducing PD-L1 expression, thereby potentiating immune checkpoint therapy. Hybrid organic-inorganic nanomaterial PB-Mn/OVA NE enhances anti-tumor effects by reversing hypoxia within the TME and activating the cGAS-STING pathway, leading to enhanced ICD and improved anti-tumor immunity. Additionally, functional composite nanosystems such as Vipadenant-carried ASPA platform modulate immunosuppressive pathways, increasing the cytolytic activity of CTLs while Treg interference. Collectively, these nanomaterials enable a multifaceted approach to cancer treatment that integrates PTT with advanced immunotherapy strategies, significantly increasing the therapeutic potential against various cancer types.

Functional composite nanosystems enhance the synergistic effect of PTT and immunotherapy by integrating various therapeutic agents and functionalities into one platform, enabling precise targeting, controlled drug release, efficient heat generation, and robust immune activation, thereby improving treatment efficacy and minimizing side effects. For instance, multifunctional nanoparticles (GOP@aPD1) encapsulated with anti-PD1 antibodies, iron oxide, and perfluoropentane in a PLGA-PEG shell enhance melanoma treatment by synergizing PTT and immunotherapy. These nanoparticles specifically deliver anti-PD1 to the tumor site, enabling effective release and increased CD8^+^ T cell infiltration, thereby activating the immune system within the TME [85]. Similarly, a semiconducting polymeric nanoantagonist (ASPA), incorporating a NIR photothermally activatable component and an adenosine A2A receptor antagonist, facilitates precise immunometabolic cancer therapy. Under NIR-II irradiation, ASPA promotes tumor ablation, triggers antagonist release to inhibit the immunosuppressive pathway, and enhances CTL function while reducing regulatory T cell activity [86]. In addition, a novel nanoimmunotherapy using Prussian blue nanoparticle-based PTT (PBNP-PTT) and systemic anti-CD137 monoclonal antibody enhances melanoma treatment. PBNP-PTT induces ICD and boosts antigen presentation, achieving tumor-free survival and reducing secondary tumor growth in vivo [87]. Moreover, an innovative in situ vaccination strategy utilizes gold nanocage-based photothermal effects, CpG oligodeoxynucleotides, and JQ1 to counteract the immunosuppressive TME. This approach effectively activates DCs, primes T cells, enhances CTL infiltration, and repolarizes macrophages towards an anti-tumor phenotype (Table 2) (Fig. 4) [88].

**Table 2:** Nanoplatforms for enhancing the synergistic effect of PTT and immunotherapy with nanotherapeutic platforms.

Nanoplatform	Composition	Mechanisms	Immune effects	Outcomes	Ref.
rGO/MTX/SB	Mitoxantrone (MTX), TGF-βinhibitor, reduced graphene oxide (rGO)	Providing an immunogenic antigen source	Infiltration of tumor-specific CTLs↑, infiltration of Tregs↓	Destroying local primary tumors and inhibiting distant metastases in 4T1 mouse mammary tumor model	[70]
PDMN-JQ1	JQ1, polydopamine nanoparticles (PDMNs)	Down-regulating the expression of PD-L1, inhibiting the BRD4-c-MYC axis	CTLs↑, immune-memory effect↑	Inhibiting TNBC in mice	[71]
CINPs	/	Activating MAPK signaling pathway, exhibiting high photothermal effect	Proportion of M1 macrophages↑, recruitment of CTLs↑	Reducing primary tumor growth and lung metastasis	[72]
Ti_3_C_2_-PEG-OVA-Mn^2+^(TPOM)	Titanium carbide Mxene, Mn^2+^-contained ovalbumin (OVA)	Activating innate immunity via the cGAS-stimulator of interferon genes signaling pathway	DC maturation↑, CTLs infiltration ↑	Enhancing antimetastasis tumor therapy	[73]
PB-Mn/OVA NE	OVA, Prussian blue (PB), manganese (Mn)	Producing oxygen to alleviate tumor hypoxic microenvironment, activating the cGAS-STING pathway	DC maturation and antigen cross-presentation ability↑, CTLs↑, memory T lymphocytes↑	Inhibiting tumors	[74]
APNA	Immunostimulant, NIR-II semiconducting transducer	Mediating photothermal effect	CTLs↑, helper T cell infiltration in distal tumor, lung and liver↑	Inhibiting cancer metastasis	[75]
IMOFH	Mesoporous organosilica nanoparticles (MONs), Fe^3+^, HA, ICG	Resulting in Fe^3+^-mediated oxidative cell death, enhancing DAMPs	Maturation of DCs↑, infiltration of CD8^+^ T cells↑	Eliminatig TNBC tumors	[76]
CEG	Cerium oxide (CeO_2_), gold	Releasing heat, forming ROS, combining with PD-1 antibody	CTLs infiltration↑	Exhibiting anti-TNBC effect in xenograft mouse models	[77]
Supramolecular cationic gold nanorod	CRISPR/Cas9 targeting PD-L1, supramolecular cationic gold nanorod	Inducing ICD and gene expression of Cas9	Conversion of DCs to T cells↑, infiltration of CTLs↑	Inhibiting the activity of primary and metastatic tumors	[78]
Au@Pt-LMDP	Au@Pt, peptide (LyP-1-PLGVRG-DPPA-1, LMDP)	Mediating photothermal effect, blocking PD-L1	Stimulating activation of CTLs↑	Eliminating primary tumors, inhibiting growth of distal tumors and alleviating tumor metastasis	[79]
AuPtAg-GOx	GOx, AuPtAg	Suppressing the production of heat shock proteins, improving the GOx activity, combining with PD-L1 antibody	CTLs↑, immunogenic "cold" TME↓	Killing cancer cells	[80]
GOP@aPD1	Anti-PD1 antibody, iron oxide, perfluoropentane, poly ethylene glycol (PEG), Gly-Arg-Gly-Asp-Ser (GRGDS) peptides, lactic-co-glycolic acid (PLGA) shell	Blocking PD1	CD8^+^ T cell infiltration↑	Enhancing antitumor efficacy	[85]
ASPA	Vipadenant, NIR-II light-absorbing semiconducting polymer	Inducing tumor thermal ablation, blocking the immunosuppressive adenosinergic pathway	CTLs functions↑, Tregs activities↓	Inhibiting primary tumor and pulmonary metastasis, forming long-term immunological memory	[86]
PBNP-PTT with CD137	Prussian blue nanoparticle (PBNP), anti-CD137 monoclonal antibody	Upregulating markers associated with antigen presentation and immune cell co-stimulation	DCs↑, tumor-infiltrating CD8^+^ T cells↑, CD4^+^and CD8^+^ T cell memory↑	Eliminating primary melanoma tumors in vivo, yielding long-term tumor-free survival.	[87]
AuNC-CpG-JQ1	Gold nanocage (AuNC), CpG, JQ1	Inducing tumor antigen production via photothermal ablation, amplifying immune responses, suppressing PD-L1	DCs↑, primed T cells↑, CTLs infiltration↑, repolarizing TAMs from M2 to M1 phenotype↑	Exhibiting a high therapeutic efficacy in the melanoma-bearing mice	[88]
*E. coli*@Cu_2_O hybrid	*E. coli*, Cu_2_O nanoparticles	Convert Cu_2_O to Cu_x_S by consuming the endogenous H_2_S, inactivating glutathione peroxidase 4 and aggregating dihydrolipoamide S-acetyltransferase	Maturation of DCs↑, activating T cells↑	Achieving tumor-specific photothermal-enhanced ferroptosis/cuproptosis and immunosuppression reversion	[81]
Triple immune nanoactivators decorated with bacteria	Polydopamine nanoparticles, tumor-specific antigens, checkpoint blocking antibodies,	/	Pro-inflammatory phenotype TAMs↑, maturation of DCs↑	Achieving potent antitumor effects in two antigen-overexpressing tumor models	[82]

Nanotherapeutic platforms combining PTT and immunotherapy offer a transformative approach to cancer treatment, achieving precise tumor ablation, immune activation, and modulation of the TME. These systems—spanning organic, hybrid, metal-based, and bacterial composite nanosystems—efficiently harness thermal effects to enhance CTL responses, promote immune memory, and convert immunologically "cold" tumors into responsive ones. The integration of PTT-specific mechanisms provides a unique pathway to potent, adaptable cancer immunotherapy, demonstrating promise for both localized and systemic tumor control.

## 6 Enhancing the synergistic effect of thermotherapy and immunotherapy with nanotherapeutic platforms

In addition to PTT, various nanomaterials have been utilized to enhance the combined effects of thermotherapy and immunotherapy. For instance, a novel metal-organic framework nanoamplifier facilitates microwave-enhanced thermotherapy and immunotherapy by targeting mitochondria to release nitric oxide and programmatically upregulating HSP70. This multifaceted approach activates CTLs, enhancing antitumor immunotherapy with a significant tumor inhibition rate [89]. Additionally, biodegradable poly (beta-amino ester) nanoparticles encapsulating mRNA for co-stimulatory molecules and cytokines, combined with a thermoresponsive gel, locally reprogram the TME. This strategy enhances lymphocyte recruitment and activation, synergizing with ICB to induce significant tumor regression and resistance to rechallenge [90]. Notably, a novel nanocatalytic modality, MNP-RGD-TPP, harnesses magnetic hyperthermia to target tumor-associated bacteria, inducing oxidative damage via a Fenton reaction. This activity not only disrupts bacteria, releasing lipopolysaccharides that polarize macrophages and mature DCs, but also promotes a robust immune response against tumors [91]. Moreover, ferrimagnetic vortex-domain iron oxide nanorings (FVIO) mediate mild magnetic hyperthermia to enhance ICD in breast cancer cells, marked by increased calreticulin expression and subsequent phagocytosis by the immune system. This strategy boosts CTL infiltration and synergizes with PD-L1 checkpoint blockade, reducing metastasis and suppressing MDSCs [92]. Nanomaterial-based thermotherapy enhances immunotherapy by inducing immune activation, modulating the TME, and increasing checkpoint blockade sensitivity. These versatile approaches provide a powerful strategy for achieving robust, localized, and systemic antitumor effects.

## 7 Enhancing the synergistic effect of chemotherapy and immunotherapy with nanotherapeutic platforms

### 7.1 Organic-based nanomaterials

Organic-based nanomaterials are effective in enhancing the combinative effect of chemotherapy and immunotherapy due to their ability to be precisely engineered for targeted drug delivery, controlled release in response to the TME, and capacity to modify surface functionalities for improved biocompatibility and immune system engagement. For instance, a novel triblock copolymeric micelle (NanoPCPT + PIMDQ) efficiently delivers chemotherapeutic and immunotherapeutic agents directly to tumors, enabling deep penetration and controlled release responsive to the tumor's acidic and reductive environment. This strategy synergistically activates DCs, enhances CTL infiltration, and mitigates regulatory T cell and M2 macrophage immunosuppression, promisingly advancing chemoimmunotherapy effectiveness [93]. Similarly, amphiphilic block copolymers, TfR-T12-PEG-PLGA and TATH7-PEG-PLGA, self-assemble into nanocomposite micelles to co-deliver PTX and imiquimod, targeting glioma via TfR-mediated Blood-Brain Barrier penetration and pH-sensitive mechanisms. These micelles modulate the TME, notably enhancing M1 polarization of TAMs and CD8^+^ T cell activity, contributing to effective glioma treatment through a synergistic chemotherapy and immunotherapy approach [94]. In addition, cisplatin-loaded poly (l-glutamic acid)-graft-methoxy poly (ethylene glycol) nanoparticles (CDDP-NPs) enhance antitumor CD8^+^ T cell responses in tumor-bearing mice by maintaining persistent tumor MHC-I overexpression, facilitating MHC-I-TCR interaction, and activating TCR signaling. Combined with agonistic OX40 antibody, CDDP-NPs significantly suppress tumor growth, underscoring their potential in synergistic cancer immunotherapy [95]. Moreover, a TME-activable prodrug nanoparticle effectively co-delivers a PD-L1 antagonist and doxorubicin (DOX) for enhanced chemoimmunotherapy in solid tumors. This nanoparticle, featuring PEGylated DOX and a D-peptide antagonist, promotes deep tumor penetration and releases therapeutic agents in situ. It simultaneously kills tumor cells, enhances CTL infiltration and reduces Tregs [96]. Furthermore, a novel nanovaccine formulation, employing redox-responsive polymer micelles encapsulating DOX and TLR7/8 agonist R848, triggers ICD and initiates an immune response. Combined with A2AR antagonist SCH58261, this approach suppresses the immunosuppressive adenosinergic pathway, enhances NK and CD8^+^ T cell activation, and inhibits regulatory T cell proliferation, thereby generating a robust antitumor immune response [97]. Significantly, a pH-responsive polyhydralazine nanoparticle (PHDZ/BTZ) effectively delivers bortezomib (BTZ) into tumors, utilizing hydralazine-induced vessel dilation to overcome transport barriers within the TME. Triggered release in acidic conditions enhances BTZ accumulation and efficacy, significantly reducing tumor growth and lung metastasis while minimizing side effects. The nanoparticle also promotes ICD, boosting CTL infiltration and proinflammatory cytokine secretion [98]. Similarly, the pH-responsive PAG/BTZ nanocarrier, modified with aminoguanidine, enhances BTZ delivery, promoting ICD and antigen uptake by DCs. This activation leads to increased DC maturation and CTL infiltration, significantly boosting antitumor immune responses and synergizing effectively with ICB [99].

In addition to the above polymer-based materials, various types of organic-based nanocarriers have been developed to deliver chemotherapeutic agents and enhance immunotherapeutic outcomes. For instance, sialic acid-functionalized liposomes, coloaded with decitabine and triclabendazole, target tumor sites to enhance pyroptosis and CD8^+^ T cell infiltration by upregulating pyroptosis-related proteins and demethylating mRNA [100]. In addition, pH-responsive nanomicelles loaded with PTX, AMD3100, and a PD-1/PD-L1 inhibitor enhance the targeting and therapeutic outcomes in TNBC by modulating the TME. This configuration significantly reduces tumor growth and metastasis, reprograms immunosuppressive conditions, and boosts CD8^+^ T cell infiltration, thereby revitalizing ICI effectiveness [101]. Similarly, an amphiphilic peptide micelle system (Co-LMs) was designed for targeted co-delivery of CpG oligodeoxynucleotides and DOX to breast cancer tumors, providing pH and redox-sensitive drug release. Demonstrating enhanced efficacy against TNBC, Co-LMs significantly increased CTL activation and reduced metastasis [102]. Moreover, chemotherapy-induced tumor RNA nanoparticles enhance cancer immunotherapy by promoting DC maturation and boosting T cell responses. These nanoparticles, formed from RNA extracted from chemotherapy-treated cancer cells, improve ICB efficacy by increasing CD8^+^ T cell to Treg ratios and facilitating T cell infiltration within tumors. Interestingly, a novel carrier-free nanoparticle integrates DOX, melittin, and anti-TOX siRNA to modulate T-cell activity in cancer therapy. This multifunctional system enhances ICD and CD8^+^ T-cell infiltration while reducing T-cell exhaustion, effectively converting "cold" tumors into "hot" ones [103].

### 7.2 Hybrid organic-inorganic nanomaterials

**Fig. 5:**
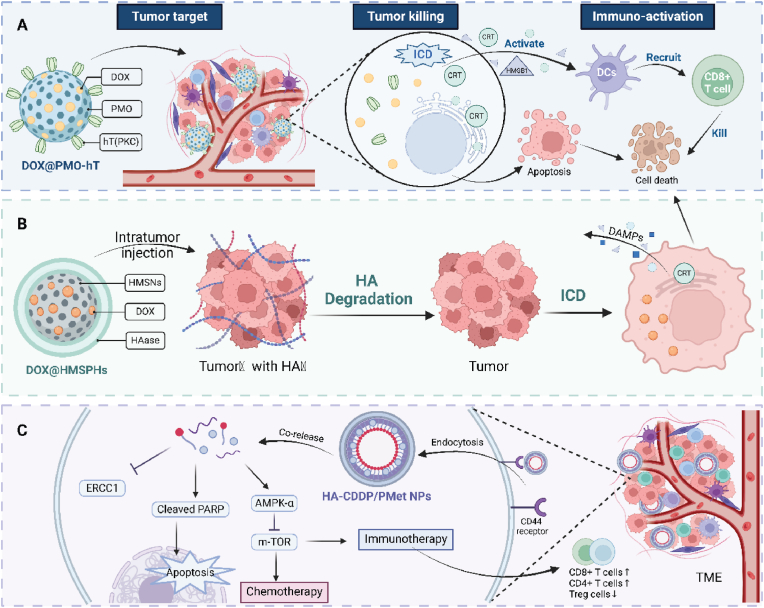
**Integration of nanomaterials to enhance the synergy of chemotherapy and immunotherapy. (A)** Periodic mesoporous organosilica nanoparticle(PMO) encapsulating DOX and hT(PKC) (a bridging molecule to attach TRAIL to the PMO nanomaterial surface) target tumor sites, where hT(PKC) improves the PMO tumor-targeting ability and promotes DOX release. This induces direct cytotoxic effects and promotes ICD, subsequently activating antigen-presenting cells and stimulating CD8^+^ T cell-mediated tumor cell killing. **(B)** Hollow mesoporous silica nanoparticles (HMSNs) encapsulating DOX and hyaluronidase are injected into tumors rich in hyaluronic acid (HA). HAase-mediated degradation of HA facilitates nanoparticle penetration and sustained DOX release, enhancing apoptosis and ICD, followed by immune responses through DAMPs and calreticulin exposure. **(C)** Nanocomplexes of cisplatin (CDDP) and metformin (Met) encapsulated in HA-coated nanoparticles target CD44 receptors on tumor cells. Co-delivery of these drugs disrupts cancer cell metabolism and DNA repair pathways, augmenting chemotherapy efficacy and promoting favorable immune modulation in the TME. This combination reduces immunosuppressive Tregs and enhances CD8^+^ and CD4^+^ T cell responses, synergistically improving overall tumor control.

Notably, various types of DOX-loaded organic-inorganic nanomaterials are utilized to enhance the efficacy of chemo-immunotherapy. Periodic mesoporous organosilica nanoparticles functionalized with TRAIL and loaded with DOX enhance antitumor immunity by targeting cancer cells and activating DCs. This dual-functional approach triggers ICD, boosts T cell activation, and demonstrates superior efficacy in suppressing tumor growth in a breast cancer mouse model, offering a promising strategy for targeted immunotherapy [104]. Similarly, DOX-loaded silica nanocarriers (DOX@HMSPHs) with hyaluronidase functionalization enhance chemo-immunotherapy by improving tumor drug permeability and inducing ICD. These nanocarriers degrade hyaluronic acid in the tumor matrix, facilitating controlled DOX release and boosting CTL activity [105]. In addition, hyaluronic acid-cisplatin/polystyrene-polymetformin (HA-CDDP/PMet) dual-prodrug nanoparticles enable synchronous delivery of cisplatin and metformin, enhancing therapeutic efficacy in lung cancer through chemo-immunotherapy. These nanoparticles improve intracellular drug delivery, synergistically inhibit tumor growth, and enhance survival in lung cancer models by modulating immune responses, increasing CD4^+^ and CD8^+^ T cells, reducing Treg cells, and activating cytokine production(Fig. 5) [106].

### 7.3 Metal-based nanomaterials

Multiple types of manganese-based nanomaterials have been developed to enhanced the combinative efficacy of chemotherapy and immunotherapy. For instance, a manganese-chelated telodendrimer nanodriver, combined with DOX nanoparticles, enhances the STING pathway in glioblastoma models, promoting DC maturation and CD8^+^ T cell infiltration [107]. In addition, amorphous porous manganese phosphate nanoparticles (PL/APMP-DOX NPs), coated with phospholipid and loaded with DOX, enhance antitumor immunity by releasing Mn^2+^ to activate the cGAS/STING pathway and DOX to induce DNA damage. These hybrid nanoparticles boost DC maturation, increase CTL and NK cell recruitment, and elevate type I interferon and pro-inflammatory cytokine production [108]. Similarly, hydrogenated mesoporous manganese oxide nanoparticles (mHMnO-Dox), camouflaged with cancer cell membranes, effectively deliver DOX and activate the cGAS-STING pathway. This dual action induces robust DC maturation, enhances CTL and NK cell recruitment, and promotes ICD, significantly inhibiting tumor growth and metastasis while prolonging survival in tumor-bearing mice [109]. Moreover, a nanoplatform (Mn-HSP) incorporating manganese ions and a PTX prodrug, based on hyaluronic acid, enhances tumor immunotherapy by inducing DNA damage and pyroptosis for tumor antigen release and activating the STING pathway. This dual mechanism promotes antigen presentation and CTL infiltration, establishing a self-perpetuating "circulating immunotherapy" that effectively suppresses primary breast tumors and metastases [110].

### 7.4 Cell- and bacteria-hitchhiking nanomaterials

Cell membranes and bacterial systems, leveraging their inherent versatility, serve as potent carriers for nanomaterials in therapeutic applications, thereby enhancing the efficacy and delivery of combined treatment strategies. For instance, cancer cell membrane-coated nanogels (ADCT@CM NGs) co-loaded with Cu (II) and toyocamycin enable targeted, redox-responsive chemodynamic and chemoimmunotherapy. These biomimetic nanogels release Cu (II) and Toy in the TME, inducing oxidative and endoplasmic reticulum stress that triggers ICD [111]. Additionally, probiotic *Bifidobacterium bifidum* modified with DOX-loaded CaP/SiO₂ nanoparticles (DNPs@Bi) combines chemotherapy and immunotherapy for enhanced anti-tumor efficacy. This bacteria-hitchhiking system achieves pH-responsive DOX release to induce ICD and promotes TAA presentation through gap junctions, enhancing DC maturation and T cell infiltration in the TME [112].

### 7.5 Functional composite nanosystems

Notably, various composite nanosystems have been employed to enhance the therapeutic outcomes of chemo-immunotherapy. For instance, LDH-PAA@DOX nanosheets, serving as both deacidification agents and chemodynamic therapy vectors, effectively neutralize the acidic TME and repolarize TAMs from M2 to M1. This transition enhances CD8^+^ T cell activation and induces significant tumor cell damage through a Fenton reaction-driven oxidative stress mechanism [113]. Interestingly, twin-like charge-switchable nanoparticles comprising shMFN1-NPs and DOX-NPs effectively target TAMs and cancer cells, respectively. These pH-responsive nanoparticles facilitate TAM repolarization from M2 to M1 phenotypes and induce ICD, enhancing DC maturation and CD8^+^ T cell activation [114]. Similarly, a charge-reversal yolk-shell liposome, co-loaded with JQ1 and DOX, enhances the stability and targeted delivery of hydrophobic drugs, effectively promoting ICD and inhibiting the PD-L1 pathway in tumors. This nanoplatform significantly boosts chemo-immunotherapy efficacy, minimizes systemic toxicity, and increases CTL infiltration [115]. In addition, a chemo-gene combinational nanomedicine, integrating DOX and siRNA within a ROS and acid-sensitive fluorine assembly, induces ICD and reverses CD8^+^ T cell exhaustion. This synergistic approach enhances immunotherapy, effectively suppressing tumor growth and metastasis in breast cancer and melanoma models [116]. Importantly, a core/shell nanoplatform integrating macrophage membrane-coated mesoporous silica nanoparticles loaded with catalase, DOX, and resiquimod enhances cancer immunotherapy. It targets tumors via ligand binding, modulates the immunosuppressive environment by catalyzing hydrogen peroxide to oxygen, blocks A2AR in Treg cells, and activates DCs [117]. Moreover, a nanoassembly, PP-(hDOX&siCD47), formed by DOX-conjugated polyphosphoester and CD47-targeting siRNA, enhances cancer immunotherapy by blocking CD47 and triggering ICD. This dual action promotes macrophage activation and T cell infiltration, reducing immunosuppressive cells in the TME and significantly inhibiting tumor growth [118]. Furthermore, a triple-combination nanosystem integrates gemcitabine prodrug nanoparticles with surface-bound anti-PD-L1 antibodies and encapsulated STING agonists, enhancing drug delivery and antitumor efficacy. This design transforms poorly immunogenic tumors into inflamed ones, inducing robust CD8^+^ T cell responses and durable tumor regression (Table 3) [119]. Nanomaterial-based platforms amplify chemo-immunotherapy by precisely targeting tumors, modulating immune responses, and inducing ICD, offering a robust approach for enhanced cancer treatment and immune activation.

**Table 3:** Nanoplatforms for enhancing the synergy between chemotherapy and immunotherapy.

Nanoplatform	Encapsulated drugs	Mechanisms	Immune effects	Outcomes	Ref.
NanoPCPT + PIMDQ	Camptothecin, TLR7/8 agonist	PH/GSH sequential response	Foxp3^+^ Tregs↓, repolarized M2 macrophages↑, CTL infiltration↑	Killing tumor cells, Inhibiting tumor growth	[93]
TfR/TATH7/PTX/R837 NMs	PTX, imiquimod	TfR-mediated BBB penetration, pH-sensitive mechanism	M1 polarization of TAMs↑, CD8^+^ T cell activity↑, TNF-α↑	Inhibiting tumor growth	[94]
CDDP-NPs	Cisplatin	Tumor MHC-I overexpression	TCR↑, costimulatory OX40 on CD8^+^T cells↑	Inhibiting tumor growth	[95]
PEG/DPPA-MMP-DOX NPs	PD-L1 antagonist, DOX prodrug, PEGylated DOX prodrug	Transcytosis process	CTLs↑, Tregs↓	Killing tumor cells, preventing tumor recurrence and metastasis	[96]
D/R@RPsP	DOX, TLR7/8 agonist R848	ICD, inhibit the immunosuppressive adenosinergic pathway	NK and CD8^+^ T cell activation↑, Tregs↓	Induction of a robust systemic antitumor immune response	[97]
PHDZ/BTZ	Hydralazine, BTZ	Dilate tumor blood vessels, Lewis acid-base coordination effect, hypoxia attenuation, ICD	CTL infiltration↑, proinflammatory cytokine secretion↑	Inhibiting tumor growth, inhibiting the occurrence of lung metastasis	[98]
PAG/BTZ nanoparticles	BTZ	pH-sensitive mechanism, ICD, promote antigen uptake	DC maturation↑, CTL infiltration↑	Enhanced antitumor efficacy	[99]
liposome modified with sialic acid (SLDT)	Decitabine, triclabendazole	mRNA demethylation, upregulate GSDME proteins, induce pyroptosis	CD8^+^ T cell infiltration↑	Enhancing targeting ability to cancer cells, Enhancing efficacy of ICB therapy	[100]
P/A/B@NM	PTX, AMD3100, PD-1/PD-L1 inhibitor	pH-responsive controlled drug release, CXCL12/CXCR4 axis blockade, ICD	CD8^+^ T cell infiltration↑	Inhibiting tumor growth and metastasis, enhancing efficacy of ICI therapy	[101]
Co-LMs	Immune adjuvant CpG, DOX	pH and redox-sensitive drug release	CTL、CD4^+^ T cell activation↑,	Inhibiting metastasis of circulating tumor cells	[102]
FD/FM@siTOX NPs	DOX, melittin, anti-TOX siRNA	ICD	CD8^+^ T cell infiltration↑, CD8^+^ T cell exhaustion↓	Converting "cold" tumors into "hot" ones	[103]
DOX@PMO-hT	DOX, TRAIL	ICD	DC、CD8^+^ T cell、CD4^+^ T cell activation↑	Inhibiting tumor growth	[104]
DOX@HMSPHs	DOX, HAase	Degradation of HA in the EM, ICD	Antigen-presentation of DCs↑, DC maturation↑, CTL activation↑	Enhancing efficacy of chemo-immunotherapy	[105]
HA-CDDP/PMet	Cisplatin, metformin	Upregulate cleaved PARP protein to induce apoptosis, induce AMPK-αpathway to inhibit mTOR, down-regulated ERCC1 protein to reduce resistance to CDDP	CD8^+^ T cells、CD4^+^ T cells↑, IFN-γ 、TNF-α ↑, Tregs↓	Inhibiting tumor accumulation and tumor growth	[106]
PLHM-DOX NPs	DOX	Activate the cGAS/STING pathway	DC maturation↑, CD8^+^ T cell infiltration↑	Inhibiting tumor growth	[107]
PL/APMP-DOX NPs	DOX	Release Mn^2+^ to activate the cGAS/STING pathway, induce DNA damage	DC maturation↑, CD8^+^ T cell infiltration↑, NK cell recruitment↑, TNF-α、IL-6↑	Enhanced antitumor efficacy	[108]
mHMnO-Dox	DOX	Activate the cGAS-STING pathway, ICD	DC maturation↑, CD8^+^ T cell infiltration↑, NK cell recruitment↑,	Inhibiting tumor growth and metastasis	[109]
Mn-HSP	PTX prodrug based on HA	Induce DNA damage and pyroptosis, activate the STING pathway	Antigen presentation↑, CTL infiltration↑	Suppressing primary breast tumors and metastases	[110]
LDH-PAA@DOX	DOX	Fenton reaction-driven oxidative stress mechanism, neutralize the acidic TME	Repolarization of TAMs to M1 phenotype, CD8^+^ T cell activation↑,	Damaging tumor cell	[113]
shMFN1-NPs + DOX-NPs(MIX-NPs)	Mitofusin 1 specific short hairpin RNA, DOX	Inhibit mitochondrial fusion to promote the conversion of macrophages, ICD	Repolarization of TAMs to M1 phenotype,DC maturation↑, CD8^+^ T cell activation↑, MDSCs↓, Tregs↓	Reversing the immunosuppressive TME	[114]
JPD@L	DOX, JQ1	Blockade PD-L1 pathway, ICD	CTL infiltration↑	Boosting chemo-immunotherapy efficacy	[115]
fPEG-fDOX@siTOX	Fluorinated DOX	Downregulate the TOX mRNA expression in CD8 T cells, ICD	Reverse CD8^+^ T cell exhaustion	Inhibiting tumor growth and metastasis	[116]
D/R/C@SiO_2_-M	DOX, R848, catalase	Hydrogen peroxide catalysis, block A2AR in Tregs, induce the ICD of tumor cells	DC maturation↑, CD8^+^ T cells↑, Tregs↓	Enhancing the antitumor immunotherapy by hypoxia reverse	[117]
hDOX&siCD47	DOX, CD47-targeting siRNA	Block CD47, ICD	M1-like macrophages↑, CD8^+^ T cell infiltration↑, MDSCs↓, Tregs↓	Inhibiting tumor growth	[118]
αPD-L1/GEM NPs	Gemcitabine prodrug, STING agonists, anti-PD-L1 antibodies	Upregulate PD-L1 expression, induce cancer cell apoptosis, activate the STING pathway,	DC maturation↑, CD8^+^ T cell response↑	Transforming low-immunogenic tumors into inflamed ones, durable tumor regression	[119]

## 8 Enhancing the synergistic effect of non-chemotherapeutic drugs or traditional Chinese medicine and immunotherapy with nanotherapeutic platforms

In addition to the traditionally used chemotherapeutic drugs mentioned above, various non-chemotherapeutic drugs and extracts from traditional Chinese medicine have been loaded into nanomaterials to enhance the efficacy of immunotherapy. A bionic nanoparticle (CP@CM), coated with activated murine vascular endothelial cell membrane and loaded with the PGE2 inhibitor celecoxib, targets postoperative melanoma to prevent recurrence. By adhering to inflammatory white blood cells and leveraging their natural tropism, CP@CM effectively delivers celecoxib to inhibit COX-2, reduce immunosuppressive cell recruitment, and increase CD8^+^ and CD4^+^ T cell infiltration at the tumor site, reversing the immunosuppressive microenvironment and enhancing postoperative immunotherapy outcomes [120]. Additionally, engineered "Gemini nanoimmunoregulators" utilize mesoporous polydopamine nanovectors to co-deliver metformin and resiquimod, wrapped in red blood cell membranes with specific peptides, to cancer cells and tumor-associated macrophages. These nanoregulators amplify ICD, reprogram macrophage phenotype from M2 to M1, degrade PD-L1, and enhance CTL function, significantly reducing tumor growth and preventing metastasis [121]. Similarly, self-degradable PMI nanogels loaded with imiquimod and metformin remodel the TME by promoting DC maturation, repolarizing M2 macrophages, and reducing PD-L1 expression. This formulation enhances CD8^+^ T cell infiltration and activation, synergizing with anti-PD-1 antibodies to amplify antitumor immune responses [122]. Notably, an in-situ vaccine utilizing acid-responsive liposome-coated polydopamine nanoparticles, modified with mannose and loaded with resiquimod, targets DCs for enhanced antigen presentation. This design leverages PTT and ICD for effective DC activation, boosting CTL responses and inhibiting tumor recurrence and metastasis through long-term immune memory, offering a refined method for cancer immunotherapy enhancement [123]. Moreover, hyaluronidase-responsive biomimetic nanoparticles (mCAuNCs@HA), optimized to 150 nm for superior circulation and tumor targeting, co-deliver pheophorbide A and a ROS-responsive PTX prodrug, enhancing on-demand drug release and ROS production. Incorporation of an anti-PD-L1 peptide augments this platform by mitigating the immunosuppressive tumor environment and boosting CTL activity [124]. Remarkably, an engineered manganese-based metal-organic framework, encapsulating polyphyllin I and coated with red blood cell membranes (RBC@Mn-MOF/PPI), enhances cGAS/STING-mediated immunity. This system prolongs circulation, targets suppressive TMEs, and activates immune cells, transforming "cold" tumors into "hot" ones, and effectively targeting primary and metastatic tumors [125]. Nanomaterial-based delivery of non-chemotherapeutic agents and traditional Chinese medicine extracts demonstrates a promising strategy for enhancing immunotherapy. By precisely targeting the TME, reprogramming immune cell functions, and activating CTLs, these platforms transform immune suppression into robust antitumor responses, offering an innovative route to advance cancer immunotherapy.

## 9 Enhancing the synergistic effect of RT and immunotherapy with nanotherapeutic platforms

Nanomaterials can significantly enhance the combined efficacy of RT and immunotherapy by not only modulating the TME to increase radiosensitivity but also facilitating the release of tumor antigens through ICD induced by radiation. This process enhances the tumor's visibility to the immune system, while the targeted delivery of immunomodulators further amplifies the immune response, leading to a more potent anti-tumor effect. For instance, AGuIX nanoparticles, acting as radiosensitizers, augment RT by exacerbating DNA damage in B16 tumor cells, enhancing the ICD and systemic T-cell responses. When combined with PD-1 blockade, this synergistic approach significantly increases CD8^+^ T cell infiltration and mitigates the immunosuppressive TME [126]. Similarly, biomimetic MnO_2_ nanoparticles encapsulating anti-PD-L1 (αPDL1@MnO_2_) enhance RT by overcoming hypoxia-induced resistance and reprogramming the TME. Released Mn^2+^ ions activate the cGAS-STING pathway, enhancing DC maturation and CTL infiltration, thus amplifying systemic antitumor responses and inhibiting metastases through a pronounced abscopal effect [127]. Additionally, multifunctional core-shell R837/BMS@Au8 nanoparticles, integrating Au8NCs with an R837/BMS nanocore, enhance RT by reducing X-ray dose and boosting antitumor immunity. These nanoparticles enhance X-ray absorption, activate effector T cells via antigen presentation, and inhibit the PD-1/PD-L1 pathway, effectively reducing tumor metastasis [128]. Moreover, Phy@PLGdH nanosheets, combining physcion with layered gadolinium hydroxide, enhance radiation therapy-induced ICD and boost in situ tumor vaccination. These nanosheets increase X-ray deposition and tumor penetration, leading to amplified oxidative stress and DNA damage. This mechanism primes CD8^+^ T-cell-dependent antitumor immunity, enhancing the efficacy of checkpoint blockade therapies against primary and metastatic tumors [129]. Interestingly, MGTe, a hybrid nanoplatform combining Te nanoparticles (GTe) for radiosensitization with fused tumor and bacterial membranes, enhances RT by producing ROS and inducing ICD under X-ray irradiation. This approach amplifies antitumor immunity through antigen presenting cells and T cell activation, demonstrating significant tumor control and potential as a clinical strategy for effective and reduced-side-effect cancer treatment [130]. Nanomaterial-based platforms significantly enhance the synergy of RT and immunotherapy by promoting ICD, overcoming hypoxia, and activating systemic immune responses. These approaches amplify radiosensitivity, prime antitumor immunity, and support robust T-cell activation, offering a powerful strategy to extend the therapeutic impact of RT and achieve durable cancer control.

## 10 Enhancing the synergistic effect of SDT and immunotherapy with nanotherapeutic platforms

Nanomaterials enhance the synergistic effect of immunotherapy and SDT by optimizing the delivery and activation of sonosensitizers within the TME. This targeted delivery facilitates ICD, releasing tumor antigens and ROS that prime the immune system. The enhanced immune activation not only boosts the local immune response but also increases systemic immune surveillance, leading to a more effective immunotherapeutic outcome. The PFH-Ce6 liposome@O_2_ nanodroplets enhance SDT by generating increased ROS and inducing ICD. When integrated with PD-1 blockade post-inadequate radiofrequency ablation, this treatment boosts antitumor immunity, reduces Tregs, and promotes DC maturation and T cell infiltration, significantly prolonging survival and initiating long-term immune memory in a preclinical cancer model [131]. Similarly, a biomimetic nanoplatform, Ru-TePt@siRNA-MVs, integrates Fenton-like sonosensitization, genetic immunotherapy, and oxygen-producing capabilities to enhance tumor oxygenation and provoke oxidative stress. This system triggers ICD, activates T cells, and silences the PD-L1 gene, synergistically augmenting the immune response against tumors [132]. Additionally, oxygen-carrying perfluorocarbon nanoparticles (M@P-SOP) enhance high-intensity focused ultrasound tumor ablation, inducing significant ICD and alleviating hypoxia in the TME. This biomimetic strategy, combined with anti-PD-L1 immunotherapy, matures DCs and repolarizes macrophages, boosting systemic antitumor responses and suppressing distant tumor growth [133]. Moreover, titanium diselenide nanosheets, utilized as nano-sensitizers under ultrasound, enhance SDT by generating ROS under both hypoxic and normoxic conditions. This treatment triggers ICD, promoting DC maturation and T-cell activation. In combination with anti-PD-1 therapy, it suppresses primary and distant tumor growth and prevents lung metastasis [134]. Nanomaterial-enhanced SDT synergizes with immunotherapy by inducing ICD and robust oxidative stress, priming both local and systemic antitumor immunity. These platforms effectively modulate the TME, activate DCs, and enhance T-cell infiltration, offering a potent strategy for achieving sustained tumor control and preventing metastasis.

## 11 Enhancing the synergistic effect of multi-modal therapies and immunotherapy with nanotherapeutic platforms

Enhancing the synergistic effect of multiple therapeutic modalities and immunotherapy using nanotherapeutic platforms involves integrating at least two conventional treatments—such as chemotherapy, RT, targeted therapy, PDT, or PTT—with immunotherapy. This approach leverages the strengths of each method to improve overall therapeutic efficacy, targeting tumor cells more effectively and stimulating a robust immune response to combat cancer. For instance, erythrocyte membrane-encapsulated copper indium selenium nanomaterials (RNCIS) harness chemodynamic, photodynamic, and photothermal mechanisms for enhanced T lymphocyte recruitment and macrophage M1 polarization. These features, combined with NIR-triggered release of NLG919, potentiate targeted photoimmunotherapy against primary and metastatic tumors [135]. Similarly, the nanoagent Cu9S5@mSiO2-PpIX@MnO2@CpG (CSPM@CpG) integrates phototherapy and immunotherapy for enhanced cancer treatment. This strategy leverages the intracellular delivery of CpG to stimulate CTLs, boost IFN-γ production, and elevate immune response levels. The synergistic effect of PTT, PDT, and immunotherapy provided by CSPM@CpG not only suppresses tumor growth but effectively inhibits metastasis, offering a potent approach to mitigate the spread of cancer [136]. In addition, supramolecular nanomicelles PolyMN-TO-8, constructed from MTX-MPEG2000, NPX-2S, and TO-8, initiate pyroptosis-mediated ICD, enhance DC maturation, and boost CD8^+^ T cell infiltration into tumors. Leveraging laser-triggered photodynamic and photothermal therapies alongside chemotherapy, these nanomicelles significantly reduce immune escape and tumor metastasis [137].

The BHMDI nanoplatform, a manganese oxide-crosslinked albumin/hyaluronic acid construct co-loaded with DOX and indocyanine green, enhances PDT for HCC. This approach alleviates tumor hypoxia, downregulates M2 macrophages, and induces ICD, boosting DC maturation and T cell activation. Coupled with PD-1 blockade, it effectively eliminates primary tumors and prevents recurrence and metastasis by invigorating systemic antitumor immunity [138]. In addition, an albumin-based nanoplatform co-delivers IR780, NLG919 dimer, and tirapazamine, enhancing PDT and chemotherapy under hypoxic conditions. This dual action activates tumor-specific CTLs and reduces immunosuppression, promoting effective intratumoral CTL infiltration and nearly eliminating tumor recurrence and metastasis in breast cancer [139]. Moreover, the iron oxide nanoparticle (FGR) integrates photothermal and chemodynamic therapies with a dextran-conjugated Toll-like receptor agonist (R848) for targeted cancer immunotherapy. Upon exposure to the acidic TME, FGR releases R848, enhancing DC maturation and CTL infiltration. This activation, coupled with direct tumor cell killing via photothermal and chemodynamic effects, effectively suppresses melanoma growth and metastasis [140]. Notably, integrating the αOX40 antibody, Ti_3_C_2_-MXene-Au nanocomposites deliver a multifunctional nanoplatform combining PTT and enzyme dynamic therapy, enhanced by photoacoustic and thermal imaging. These treatments induce ICD and apoptosis, promoting DC maturation and T-cell infiltration, further enhancing the efficacy of immunotherapy [141]. Furthermore, the Ag_2_S QD/DOX/Bestatin@PC_10_ARGD polypeptide hydrogel, incorporating a photosensitizer, chemotherapy drug, and immune-adjuvant, serves as a multifunctional platform for mammary carcinoma treatment. Triggered by PTT, this hydrogel facilitates sustained drug release, enhances immune cell infiltration, and significantly inhibits tumor growth and metastasis [142]. Interestingly, LINC, a light-inducible nanocargo, integrates a photosensitizer and an IDO-1 inhibitor with a light-activatable prodrug of oxaliplatin, enhancing chemoimmunotherapy by ensuring targeted delivery and controlled drug release in tumor sites. This dual-light-triggered system enhances deep tumor penetration and retention, facilitating increased CTL infiltration and reducing immunosuppression within the TME [143].

**Fig. 6:**
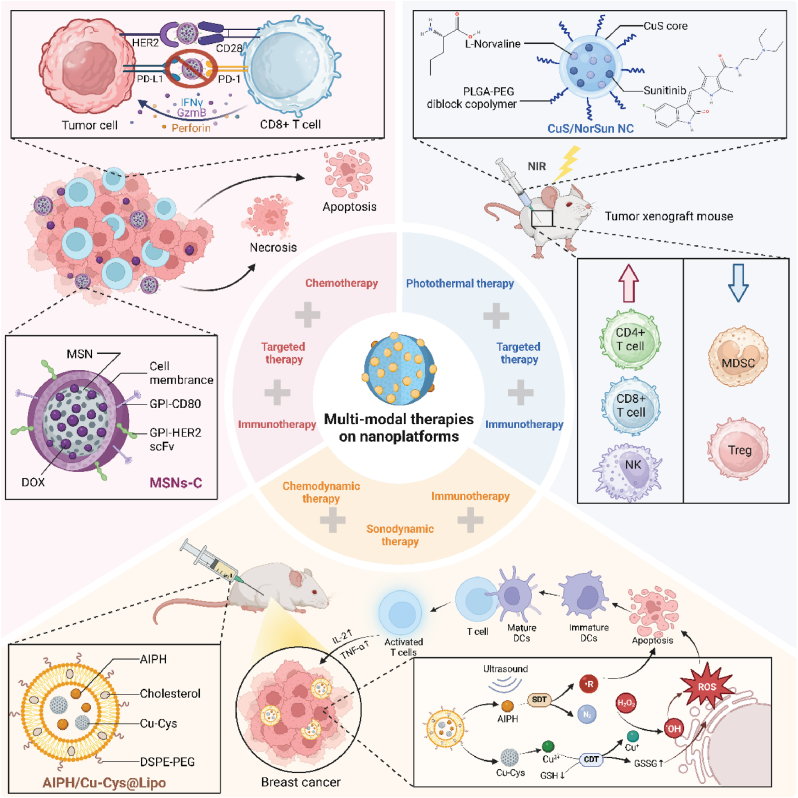
**Nanomaterials facilitate synergistic multimodal therapies in cancer treatment.** Nanomaterials significantly enhance the synergistic effects of conventional therapies and immunotherapy by integrating multiple therapeutic modalities on a single platform. Mesoporous silica nanoparticles (MSNs) are engineered to deliver DOX while presenting cell membrane proteins and targeted antibodies like GPI-CD80 and HER2 scFv, enhancing targeted therapy and immunogenic response. These MSNs facilitate targeted drug delivery directly to tumor cells, promoting apoptosis and necrosis. Additionally, copper sulfide nanoparticles (CuS/NorSun NC) incorporate L-norvaline and sunitinib, affecting tumor metabolism in a tumor xenograft mouse model. This combination modulates the TME, influencing the proliferation of CD4^+^ T cells and the activity of MDSCs and Tregs, promoting a more effective immunotherapeutic response. Further, lipid-coated nanoparticles (AIPH/Cu-Cys@Lipo) use ultrasound and reactive oxygen species generation to induce apoptosis in breast cancer cells, enhancing sonodynamic and chemodynamic therapies. Collectively, these nanoplatforms orchestrate a comprehensive and targeted attack on cancer cells, significantly amplifying the efficacy of combined therapeutic strategies and leading to improved treatment outcomes.

In addition to the above-mentioned phototherapy-involved multi-modal therapies, targeted therapy and SDT-involved multi-modal therapies also improve immunotherapy outcomes. These integrated approaches enhance the therapeutic efficacy by leveraging the strengths of each method, ultimately boosting the immune response against cancer. For instance, a novel biomimetic nanoparticle system enhances solid tumor therapy by integrating chemotherapy, targeted therapy and immune synergy. Mesoporous silica nanoparticles, loaded with DOX and coated with cell membranes featuring GPI-anchored anti-HER2 scFv and CD80, effectively increase tumor targeting and immune response activation. This system improves antitumor efficacy by boosting CD8^+^ T cell activity, enhancing cytokine production, and reducing suppressive cell populations in tumor environments [144]. In addition, biocompatible nanocarriers targeting MDSCs were developed to combat immunosuppressive chemoresistance in lung cancer. These carriers deliver l-Norvaline and Sunitinib, enhancing apoptosis, reducing tumor volume, and increasing drug retention. They significantly boost CD8^+^ and CD4^+^ T cell infiltration, activate NK cells, and reduce Treg cells and MDSCs in the TME. Enhanced tumor ablation under NIR exposure underscores the potential of this strategy for effective metastatic cancer treatment [145]. Interestingly, AIPH/Cu-Cys@Lipo, a sonodynamic-chemodynamic liposome, effectively depletes glutathione, generating radicals via ultrasound and -OH through Fenton-like reactions. This nanoparticle enhances tumor immunotherapy by disrupting mitochondrial function and activating CD4^+^ and CD8^+^ T cells, thereby increasing IL-2 and TNF-α expression, and significantly inhibiting tumor growth (Table 4) (Fig. 6) [146]. Multi-modal nanotherapeutic platforms integrating phototherapy, chemotherapy, targeted therapy, and SDT with immunotherapy offer a comprehensive strategy to enhance antitumor immunity. By harnessing distinct mechanisms—such as ICD, immune modulation, and precise tumor targeting—these platforms amplify immune responses, reduce tumor escape, and inhibit metastasis. This approach represents a powerful advancement for durable and highly effective cancer treatments.

**Table 4:** Nanoplatforms for synergistic multimodal therapies in cancer treatment.

Nanoplatform	Composition	Therapeutic modalities	Immune effects	Therapeutic efficacy	Ref.
RNCIS NPs	NLG919, CuInSe_2_, DSPE-mPEG, erythrocyte membrane,	PTT, PDT, chemodynamic therapy, immunotherapy	CTL recruitment↑, macrophage M1 polarization↑	Killing cancer cells, eliminating primary and metastatic tumors	[135]
CSPM@CpG	Cu9S5, mSiO_2_, PpIX, MnO_2_, CpG	PTT, PDT, immunotherapy	CTL infiltration↑, IFN-γ↑	Inhibiting tumor growth and metastasis	[136]
PolyMN-TO-8	MTX-MPEG2000, NPX-2S, TO-8	PTT, PDT, chemotherapy, immunotherapy	DC maturation↑, CD8^+^ T cell infiltration↑	Reducing immune escape and tumor metastasis	[137]
BHMDI	DOX, indocyanine green, hyaluronan, MnO_2_-crosslinked bovine albumin	PDT, chemotherapy, immunotherapy	DC maturation↑, T cell activation↑	Alleviating tumor hypoxia, eliminating primary tumors, preventing tumor recurrence and metastasis	[138]
IR780-NLG919-TPZ NPs	IR780, NLG919 dimer, tirapazamine	PDT, chemotherapy, immunotherapy	CTL infiltration↑	Suppressing primary and distant tumors, inhibiting tumor recurrence and metastasis	[139]
FGR	Iron oxide, R848	PTT, chemodynamic therapy, immunotherapy	DC maturation↑, CTL infiltration↑	Killing tumor cells, suppressing melanoma growth and metastasis	[140]
Ti_3_C_2_-MXene-Au	OX40 mAb, AuNP, Ti_3_C_2_-MXene	PTT, enzyme dynamic therapy, immunotherapy	DC maturation↑, T cell activation↑	Destroying cancer cells, reversing the immunosuppressive microenvironment	[141]
Ag_2_S QD/DOX/Bestatin@PC10ARGD hydrogel	Ag_2_S quantum dot, DOX, Bestatin,	PTT, chemotherapy, immunotherapy	DC maturation↑, CD8^+^ T cell infiltration↑,IL-12p70↑, IFN-γ↑	Inhibiting tumor growth and metastasis	[142]
LINC	Pheophorbide A, NLG919, light-activatable prodrug of oxaliplatin	PTT, chemotherapy, immunotherapy	CTL infiltration↑	Inhibiting the tumor growth and recurrence, inhibiting lung metastasis	[143]
MSNs-C	DOX, anti-HER2 scFv- and CD80-modified cell membranes	Chemotherapy, targeted therapy, immunotherapy	CD8^+^ T cell activation↑, MDSCs↓	Inhibiting the growth of implanted breast tumors	[144]
CuS/NorSun NCs	l-Norvaline, Sunitinib	PTT, immunotherapy, targeted therapy,	CD8^+^ and CD4^+^ T cell infiltration↑, NK cell activation↑, MDSCs↓, Tregs↓	Enhancing tumor ablation	[145]
AIPH/Cu-Cys@Lipo	2,2-azobis[2-(2-imidazolin-2-yl) propane] dihydrochloride (AIPH), copper-cysteine, liposome	Chemodynamic therapy, SDT, immunotherapy	CD8^+^ and CD4^+^ T cell activation↑, IL-2↑, TNF-α↑	Inhibiting tumor growth	[146]

## 12 Comparative analysis of nanoplatforms

A comparative analysis of nanoplatforms for cancer therapy reveals that each type offers distinct structural and physicochemical properties, which contribute to their specific therapeutic outcomes and versatility in clinical applications. Organic-based nanomaterials, such as polydopamine and graphene oxide, benefit from high biocompatibility and inherent biodegradability due to their organic molecular structures, making them ideal for tumor-specific targeting with minimal systemic toxicity. Their structure allows efficient functionalization, enabling PTT and immune activation that stimulate CTL responses while suppressing Tregs to control tumor growth [147,148]. Hybrid organic-inorganic nanomaterials, by integrating organic components with inorganic elements, exhibit improved stability and multifunctionality. This structure supports potent PDT effects while enabling hypoxia reversal in the TME and facilitating immune cell infiltration, achieving superior tumor ablation compared to single-modality therapies [149].

Metal-based nanomaterials, including copper sulfide and iron oxide nanoparticles, offer unique magnetic and optical properties due to their metallic composition. Their structures are conducive to photothermal effects and ROS generation, which disrupt hypoxic TME barriers and enhance drug delivery precision. These properties not only amplify tumor suppression but also improve specificity in immune activation. MOF-based nanomaterials leverage their highly porous structures, allowing high drug loading and controlled release of therapeutic agents. These structural characteristics enhance PDT efficacy and enable sustained activation of cytotoxic immune responses, leading to better survival outcomes in preclinical models while minimizing systemic side effects [150]. Cell- and bacteria-hitchhiking nanomaterials, leveraging living carriers such as cells or bacteria, enhance the targeted delivery and retention of therapeutic agents in the TME through active homing mechanisms. These systems provide a natural biocompatible shield, improving circulation time and reducing immune clearance while facilitating precise delivery to tumor sites. Additionally, their ability to penetrate tumor tissues and modulate the TME amplifies therapeutic efficacy [151].

Lastly, functional composite nanosystems combine various therapeutic agents—including chemotherapeutic, immunomodulatory, and photothermal components—on a single, multifunctional platform. The composite nature of these systems allows for precise targeting, controlled drug release, and simultaneous engagement of multiple pathways within the TME, maximizing synergistic effects. Their robust and adaptable structure has demonstrated an exceptional capacity to reduce tumor volume and activate adaptive immunity in multiple cancer models, underscoring their significant therapeutic potential. This structural and physicochemical comparison of nanoplatforms highlights the specific material advantages each type offers, informing the selection of nanoplatforms for targeted, effective multimodal cancer therapies.

## 13 Challenges in nanoplatform applications for cancer immunotherapy

The convergence of nanotherapeutic platforms with traditional cancer therapies, such as PDT, PTT, chemotherapy, RT and SDT, alongside immunotherapy, represents a promising frontier in oncology. By exploiting the unique properties of nanoparticles, these combined approaches aim to enhance the precision and potency of cancer treatment modalities. Nanoparticles can specifically deliver therapeutic agents to tumor sites, modulate drug release rates, and even actively target TME, thereby amplifying the therapeutic efficacy and minimizing systemic toxicity [152]. Additionally, nanoparticles can be engineered to carry both cytotoxic drugs and immunomodulatory agents, facilitating a dual attack on cancer cells while simultaneously priming the immune system [153]. This synergistic strategy harnesses the strengths of each therapy, potentially overcoming the limitations of each when used alone. However, despite these promising advances, significant challenges remain.

Firstly, current research on nanomaterials predominantly focuses on their characterization under in vitro conditions, with only a limited number of studies venturing into in vivo evaluations. However, these in vivo studies are primarily constrained to small animal models, particularly mice [[154], [155], [156]], which may not accurately replicate human physiological responses due to differences in scale, immune system characteristics, and metabolic rates [157]. This limitation significantly hampers the translation of nanomedical innovations from the laboratory to the clinic. Future investigations need to extend beyond rodent models to include larger animal models, such as pigs or non-human primates, which offer more comparable anatomical and physiological traits with humans. These models could provide more relevant data on the biodistribution, pharmacokinetics, and long-term biocompatibility of nanomaterials, thereby enhancing the predictability of clinical outcomes [158,159]. Such comprehensive animal studies are crucial for advancing nanomedicine, enabling more effective and safe therapeutic solutions that can be confidently moved into human trials. This expansion in model diversity will also contribute to a better understanding of the nano-bio interactions at play, essential for optimizing the design and functionalization of nanomaterials for specific therapeutic applications.

Secondly, scaling up the production of nanotherapeutics from laboratory prototypes to industrial-scale manufacturing presents significant challenges due to the complex nature of nanomaterial designs and the precise requirements for ligand modification and drug loading strategies. Standardized protocols for the characterization and production of these nanomaterials are essential to mitigate variability, which can lead to significant discrepancies in experimental and clinical outcomes, thus hindering reproducibility essential for clinical translation [160]. In response, ongoing efforts in the field are increasingly focused on developing uniform standards and optimized production methods to enhance consistency and control across the manufacturing process [161]. Moreover, it is critical that the scale-up process rigorously addresses the stability, quality, and safety of nanomaterials, ensuring each batch produced meets stringent criteria. Enhanced predictive models and quality assessment frameworks are being integrated into development workflows to systematically evaluate the long-term stability and performance of nanomaterials under various conditions [162,163]. This level of standardization is crucial not only for maintaining the integrity of therapeutic effects but also for complying with the regulatory standards set forth by health authorities such as the U.S. Food and Drug Administration (FDA) and the European Medicines Agency (EMA). In addition, there is a growing emphasis on early engagement with regulatory agencies to facilitate a smoother approval process. Proactive collaboration with regulatory bodies helps to clarify specific requirements and align nanotherapeutic development with regulatory expectations from the outset. This approach ensures that all necessary data, from manufacturing process details to quality control measures, are collected in compliance with established standards [164]. By aligning production practices and regulatory considerations early, the pathway to clinical trials and market entry is streamlined, ultimately ensuring that these innovative treatments are delivered to patients both efficiently and safely.

Thirdly, the design and application of nanoplatforms in medical therapies are primarily governed by their biodegradability, excretion, toxicity, and non-toxicity profiles, which are essential for ensuring both clinical efficacy and minimal environmental impact. Ideally, these nanoplatforms should be constructed from materials that degrade into biologically benign byproducts naturally metabolized by the body, thus preventing long-term accumulation and associated toxicity [165]. The physicochemical characteristics of nanoparticles, such as size, shape, and surface properties, critically influence their biodistribution, determining their clearance mechanisms—smaller nanoparticles are typically excreted renally, reducing the risk of bioaccumulation, whereas larger particles may require surface modifications, such as pegylation, to enhance solubility and promote excretion through the liver and bile [166]. Toxicity is a major concern, as nanoparticles can interact with cellular and subcellular structures, potentially causing oxidative stress, ion release, and other cytotoxic effects. Rigorous toxicity assessments through both in vitro and in vivo studies are essential to ensure that nanoparticles do not induce harmful biological responses such as inflammation or immune reactions. In terms of non-toxicity, it is crucial that nanoplatforms are designed to perform their therapeutic roles effectively without eliciting any adverse biological interactions. This involves engineering nanoparticles with 'stealth' capabilities that evade the immune system, prolonging their circulation time and enhancing their delivery efficiency [167].

Fourthly, combination immunotherapy based on nanomaterials, while more effective than monotherapies, introduces a greater complexity of effects, including an increased risk of autoimmune reactions, toxicity, and adverse events in patients [[168], [169], [170]]. The interplay between different therapeutic modalities leveraged in these approaches involves a myriad of potential targets and pathways, each contributing uniquely to the therapeutic outcome. Currently, understanding of the synergistic effects between these modalities is primarily superficial, with the underlying mechanisms still largely unexplored. There is a critical need to delve deeper into these interactions to determine whether antagonistic effects exist or if there could be unintended side effects on normal tissues. Further, while preliminary results are promising, rigorous clinical and mechanistic studies are essential to elucidate the specific contributions of each component in the combination therapy.

Fifthly, the development of personalized combination therapies is essential due to the inherent heterogeneity of tumors and specific genetic mutations in individual patients. Leveraging nanoplatforms, these tailored therapies can deliver targeted treatments more effectively [171]. Nanoplatforms can be engineered to transport multiple therapeutic agents, specifically targeting tumor characteristics and patient-specific genetic profiles, thereby enhancing precision and efficacy while minimizing side effects. Notably, advanced genomic and proteomic technologies are pivotal in identifying optimal therapeutic combinations and treatment sequences that nanoplatforms can facilitate [172]. Additionally, the ability to monitor treatment responses in real time and dynamically adjust strategies is crucial for effective personalized therapy. By utilizing nanoplatforms in a patient-centric treatment approach, it not only promises enhanced disease management but also supports the broader objectives of precision medicine, aiming to tailor medical decisions and treatments to individual patient needs.

Lastly, the economic implications of implementing nanoplatform-based cancer therapies are significant and multifaceted, with potential long-term impacts on healthcare systems. The development and production of these therapies entail high costs due to complex manufacturing processes, quality control, and regulatory compliance. These factors can translate to high treatment costs, raising concerns about affordability and accessibility. For healthcare systems, this cost structure may necessitate increased budget allocations or adjustments in resource distribution, potentially impacting the provision of other services [173]. However, nanoplatform-based therapies offer benefits in terms of treatment precision and reduced adverse effects, which could lower downstream costs associated with managing treatment side effects, hospital readmissions, and lengthy recovery times. As production methods advance and standardization improves, the cost efficiency of these therapies may increase, which could alleviate some economic pressures [174]. Overall, a detailed economic assessment is essential to evaluate the cost-benefit balance and inform policy decisions on integrating nanoplatform-based therapies within healthcare systems, ensuring sustainable and effective implementation.

## 14 Ongoing and future developments in nanotherapeutics

The field of nanotherapeutics is experiencing rapid advancements, driven by cutting-edge research and technological innovations. A significant area of development is in the engineering of stimuli-responsive nanoparticles, designed to react to specific environmental cues within the TME, such as pH fluctuations or the presence of specific enzymes. These advanced materials are tailored to optimize the targeted delivery and controlled release of therapeutic agents, enhancing treatment precision while mitigating collateral damage to healthy tissues [152,175,176]. Concurrently, the integration of computational tools, notably artificial intelligence (AI) and machine learning (ML), is transforming nanomedicine [177]. These technologies are instrumental in refining the design and functionality of nanoparticles, allowing for the simulation and prediction of nanoparticle behavior in complex biological systems [178,179]. By leveraging vast datasets to forecast interactions at the molecular level, AI and ML are facilitating the creation of nanoparticles customized to the genetic and molecular profiles of individual patients. This approach not only enhances therapeutic efficacy but also ushers in a new paradigm of personalized medicine, where treatments are specifically tailored to the unique characteristics of each patient's disease. Moreover, the development of theranostic nanosystems represents a convergence of diagnostic and therapeutic modalities into a single platform [180]. These multifunctional systems are designed to detect cancer at its earliest stages, monitor the dynamic responses of tumors to treatments in real-time, and deliver therapeutic agents precisely where needed. By combining imaging and therapeutic capabilities, theranostics provide a powerful tool for oncologists to optimize treatment strategies, adjust dosages, and predict therapeutic outcomes with greater accuracy [181,182]. As these technological and scientific advancements progress, the need for robust interdisciplinary collaboration becomes increasingly evident. The translation of nanotherapeutics from the laboratory to the clinic necessitates close cooperation among scientists, clinicians, and regulatory agencies. Establishing comprehensive regulatory frameworks and standards for the approval and clinical use of nanotherapeutics is critical [183]. These frameworks must address the unique challenges posed by nano-scale materials, ensuring that they meet safety and efficacy criteria without stifling innovation.

## 15 Conclusions

The integration of immunotherapy with various treatment modalities, facilitated by nanoplatforms, represents a significant advancement in cancer treatment. Combining immunotherapy with traditional cancer therapies, and targeted nanomaterial applications enhances efficacy and addresses the limitations of single-modality treatments. Continued refinement of nanoplatform specificity and delivery mechanisms is essential to minimize off-target effects and toxicity. Future research should focus on patient-specific therapeutic combinations to optimize treatment outcomes. Comprehensive clinical trials are necessary to validate the safety and effectiveness observed in preclinical models and to investigate long-term outcomes and immunological effects. These efforts are critical for realizing the full potential of nanotechnology in revolutionizing cancer therapy.

## CRediT authorship contribution statement

**Rongwei Xu:** Writing – original draft, Visualization, Methodology, Investigation, Conceptualization. **Pei Lin:** Writing – original draft, Visualization, Methodology, Investigation. **Jiarong Zheng:** Writing – original draft, Visualization, Resources, Methodology, Investigation. **Yunfan Lin:** Writing – original draft, Visualization, Resources, Methodology, Investigation. **Zizhao Mai:** Writing – original draft, Visualization, Software, Methodology. **Ye Lu:** Writing – original draft, Visualization, Methodology, Investigation. **Xu Chen:** Writing – original draft, Visualization, Methodology, Investigation. **Zihao Zhou:** Writing – original draft, Visualization, Methodology, Investigation. **Li Cui:** Writing – review & editing, Supervision, Project administration, Investigation, Funding acquisition, Conceptualization. **Xinyuan Zhao:** Writing – review & editing, Visualization, Project administration, Funding acquisition, Conceptualization.

## Declaration of competing interest

The authors declare that they have no known competing financial interests or personal relationships that could have appeared to influence the work reported in this paper.

## Data Availability

No data was used for the research described in the article.
